# Extracellular vesicle‐derived microRNAs as potential biomarkers in oligoarticular juvenile idiopathic arthritis patients: methodological challenges and new perspectives

**DOI:** 10.1002/ctm2.1067

**Published:** 2022-09-30

**Authors:** Federica Raggi, Davide Cangelosi, Alessandro Consolaro, Chiara Rossi, Simone Pelassa, Katia Cortese, Maria Cristina Gagliani, Martina Morini, Daniela Segalerba, Chiara Brignole, Paola Bocca, Danilo Marimpietri, Chiara Trincianti, Angelo Ravelli, Alessandra Eva, Maria Carla Bosco

**Affiliations:** ^1^ Laboratory of Molecular Biology IRCCS Istituto Giannina Gaslini Genova Italy; ^2^ Pediatric Rheumatology Clinic IRCCS Istituto Giannina Gaslini Genova Italy; ^3^ DiNOGMI University of Genova Genova Italy; ^4^ Cellular Electron Microscopy Laboratory, Department of Experimental Medicine University of Genova Genova Italy; ^5^ Laboratory of Experimental Therapies in Oncology IRCCS Istituto Giannina Gaslini Genova Italy; ^6^ Present address: Unit of Autoinflammatory Diseases and Immunodeficiences, Pediatric Rheumatology Clinic IRCCS Istituto Giannina Gaslini Genova Italy; ^7^ Cell Factory IRCCS Istituto Giannina Gaslini Genova Italy; ^8^ Scientific Direction IRCCS Istituto Giannina Gaslini Genova Italy; ^9^ Present address: Clinical Bioinformatics Unit IRCCS Istituto Giannina Gaslini Genova Italy; ^10^ Present address: Laboratory of Experimental Therapies in Oncology IRCCS Istituto Giannina Gaslini Genova Italy

Dear Editor,

Oligoarticular juvenile idiopathic arthritis (OJIA) is the most common chronic paediatric arthritis in Western countries and a major cause of childhood disability.^1^ Early diagnosis and prediction of disease course and therapeutic response are crucial for patient management but are hindered by the lack of biomarkers. Identification of new low‐invasive biomarkers is thus highly required to improve the current diagnosis of OJIA patients and help with the setting up of targeted treatments at disease onset, which would allow to reduce the burden of the disease, limit the occurrence of joint damage and loss of functionality, minimize patient exposure to the potential side‐effects of ineffective medication, and induce earlier remission, thus enhancing patient quality of life^2^ (Supporting Information, Section 1). Extracellular vesicles (EVs) released into biologic fluids have gained increased recognition as key mediators of the pathogenesis of many chronic inflammatory and autoimmune diseases, including adult arthritides,^3^ and as a reliable and enriched source of disease biomarkers, by virtue of their cargo of bioactive molecules (nucleic acids, proteins, and lipids) of cellular origin, which can be transferred to, and elicit responses in, recipient cells.^4^ Analysis of microRNAs encapsulated in EVs (EV‐miRs) provides a powerful means for new non‐invasive biomarker discovery, given their high stability, concentration, and integrity coupled to tissue specificity^5^ (Supporting Information, Section 1). Recent studies indicated the diagnostic/prognostic potential of dysregulated EV‐miRs in adult rheumatic patients.^6^, ^7^ However, EV‐miR expression and potential as biomarkers in OJIA has not been investigated. The aim of this study was to identify candidate EV‐miR‐based biomarkers in children with newly diagnosed OJIA. Because joints are the main targets of clinical manifestations in OJIA, we first optimized the protocol for profiling EV‐miRs in small volumes of synovial fluid (SF) collected from arthritic joints. We then searched for EV‐miRs potentially implicated in disease development by assessing the EV‐miRNome of SF versus paired plasma (PL) from OJIA patients at disease onset. Finally, we derived EV‐miR signatures with diagnostic potential by comparing the EV‐miR profiles in specimens isolated from new‐onset OJIA patients versus control children (CTR).

New‐onset OJIA patients were enrolled consecutively in the study and grouped in a training (*n* = 5) and a validation (*n* = 8) cohort. Patient characteristics are reported in Table S1 and in the Supporting Information (Section 2.1). EVs were isolated from SF aspirates using membrane‐affinity spin columns and characterized in terms of recovery, dispersion, size, morphology, and the expression of typical EV markers^8^ (Supporting Information, Sections 2.2–2.7). Total RNA was extracted from EVs, and the miRNA content was assessed and profiled. Expression data were analysed as described^9^ (Supporting Information, Sections 2.8–2.10). A workflow chart is depicted in Figure S1. To establish a reliable procedure for EV‐miR profiling in SF, we evaluated the impact of two anticoagulants used for SF collection, EDTA and sodium‐heparin, and of sample pre‐treatment with hyaluronidase (HYase) to remove hyaluronic acid contaminants, which may affect EV‐miR isolation and detection.^8^ We demonstrated the efficient recovery of a relatively monodisperse population of particles with morphology, size range and marker expression consistent with both exosomes and microvesicles (Figure 1A–E).^8^ The best performance for EV‐miR detection was observed upon sample collection in EDTA combined with HYase pre‐treatment (Figure 1F). Increased EV‐miR number and expression levels were detectable in EDTA+HYase samples from both patients of the training (Figures S2, S3A, and Table S2) and validation (Figure 2A,B and Table S3) cohorts (Supporting Information, Sections 3.1–3.3), with some of the identified miRNAs (Figure S3B and Figure 2C) previously associated with the pathogenesis of, and regarded as promising biomarkers in, adult chronic arthritides.^3^, ^7^ These data highlight the suitability of our experimental protocol for the definition of potential EV‐miR‐based biomarkers in OJIA‐SF (Supporting Information, Section 4).

**FIGURE 1:**
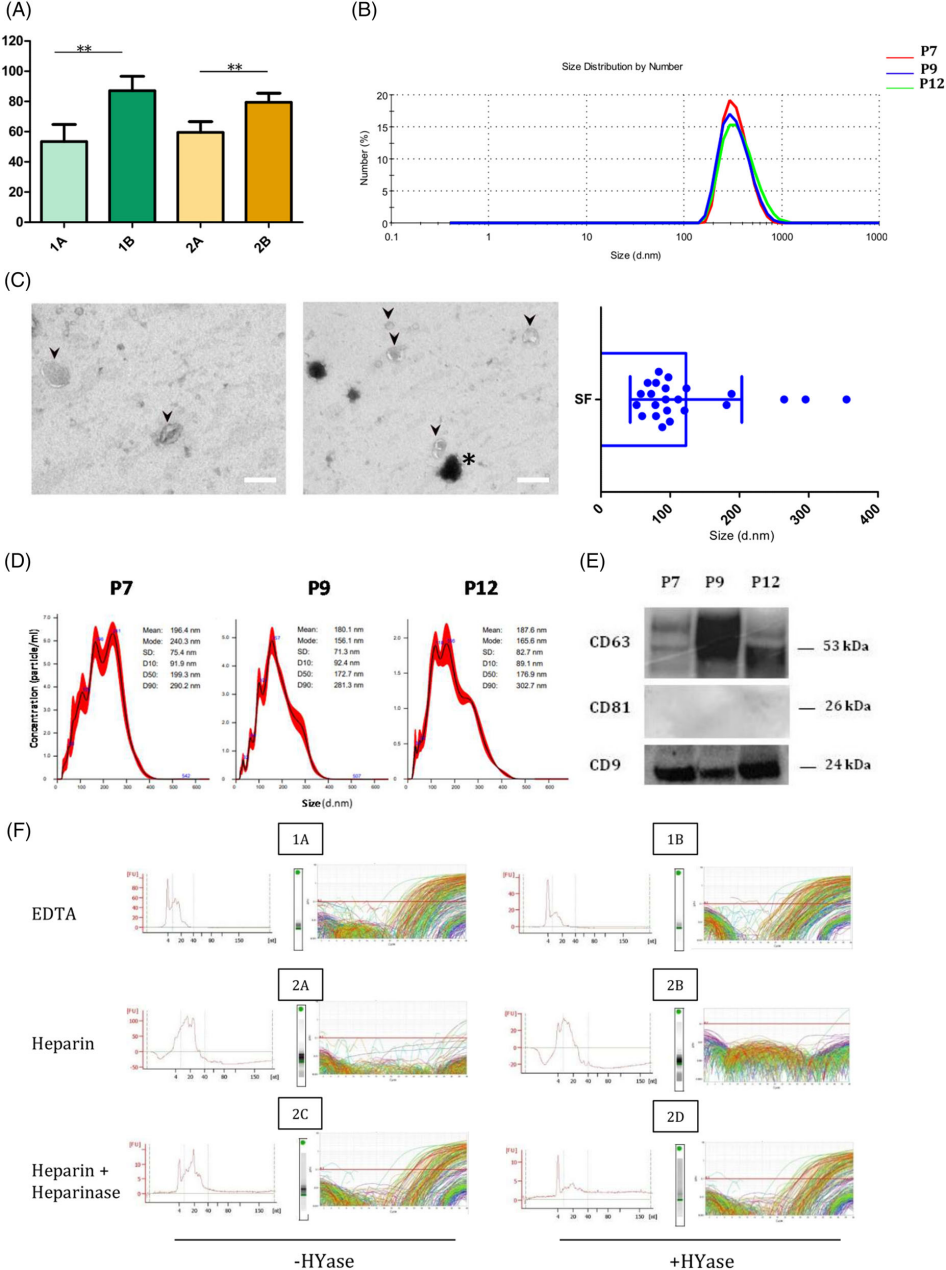
**Characterization of EVs and EV small‐RNA profiles in SF samples from new‐onset OJIA patients**. EVs were purified from 500 μl SF samples of new‐onset OJIA patients using‐a membrane affinity spin column‐based procedure and characterized. (A) The bar graph indicates the protein count of EVs isolated from samples collected in EDTA (samples 1A,1B) or sodium‐heparin (samples 2A,2B) and treated with HYase (samples 1B, 2B) or left untreated (samples 1A,2A), as detailed in the flow chart in Figure S1. Three representative samples/patients for each group were analysed. Values are expressed as mean ± SD. Statistical analysis was performed using paired Student's *t*‐test: **p < .01. (B) Curves show the size distribution profiles of EVs isolated from EDTA‐collected and HYase‐treated samples of three representative patients measured by dynamic light scattering (DLS). The diameter (nanometres, nm) of EVs is reported on the X‐axis, while the number is reported on the Y‐axis (peaks at 345, 346 and 380 nm; PDI 0.16, 0.19 and 0.20). (C) Representative transmission electron microscopy (TEM) images of isolated EVs visualized by negative staining (left and middle panels). EVs with the typical ultrastructural morphology are indicated by arrowheads. The presence of dense precipitates on some round structures, likely EVs (asterisks), could be ascribed to the reagents used in the isolation procedure. Scale bars = 200 nm. The size distribution of isolated EVs analysed by TEM is visualized as a scatter dot plot and a histogram (right panel). Each point represents the size measurement of a single EV (excluding the dark particles and small aggregates); the histogram indicates size means and SD assessed using descriptive statistic tools of the GraphPad Prism software (mean diameter 123 ± 80 nm). At least five independent images per sample were analysed and particles with a diameter ≥30 nm were measured. (D) NTA of EVs isolated from SF samples. Curves show the size distribution profiles of EVs isolated from the same patients analysed in panel B and diluted 1:500 in particle‐free PBS. Line graphs correspond to the average size of five different measurements for each sample. The diameter (nm) of EVs is reported on the X‐axis, while the concentration (particles/ml) is reported on the Y‐axis. EV size mean and mode are indicated. D90, D50 and D10 refer to the percentage of EVs (90%, 50% and 10%) with sizes lower than the indicated values. (E) Western blot analysis of EV protein extracts from the samples showed in panel B. Seven micrograms proteins were resolved on a 4–12% Tris‐Glycine Mini Gel, and the blot was hybridized with mAbs specific for the EV markers, CD9 (size 24 kDa), CD63 (differently glycosylated forms ranging between 45 and 60 kDa) and CD81 (size 26 kDa). (F) Total RNA was extracted from purified EVs and treated with heparinase or left untreated. Small‐RNA quality and miRNA content were evaluated by capillary electrophoresis using the small RNA Assay. Electropherograms of small RNAs from a representative OJIA patient are shown. The miRNA fraction is included between the dotted lines of the small RNA profile. The x‐axis reports the length of RNA molecules in nucleotides [nt]; the y‐axis shows the fluorescence units [FU] proportional to RNA concentrations. Plots represent the qRT‐PCR amplification curves of the EV‐RNA from samples of a representative patient. The threshold cycle is set at .1 (horizontal line).

**FIGURE 2:**
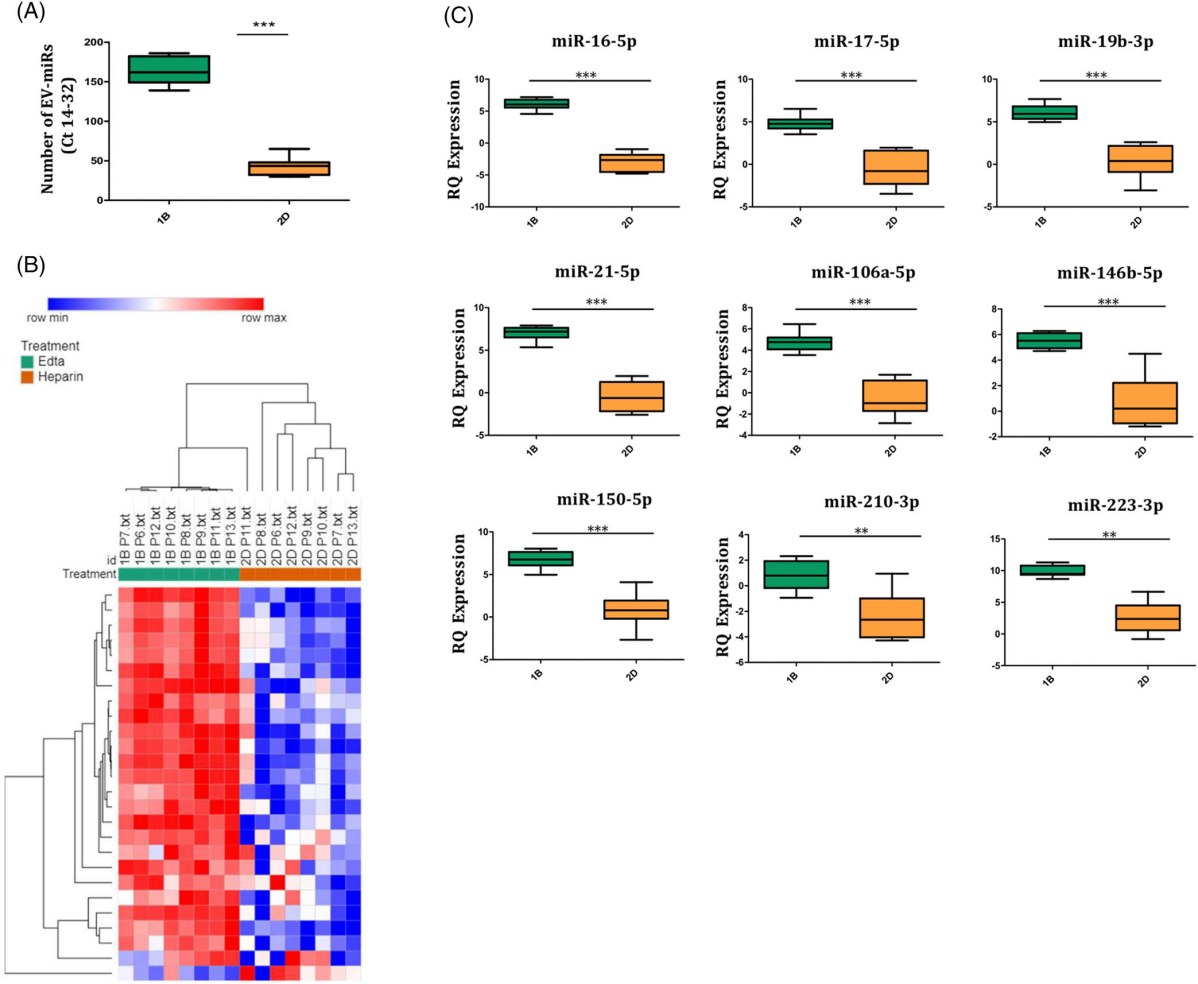
**Comparative analysis of EV‐miR expression profiles between EDTA and heparin/de‐heparinized SF samples from patients of the validation cohort** . EV‐miRs isolated from SF samples of patients of the validation cohort were profiled by the TaqMan‐Array‐Human MicroRNA A Cards, and expression data analysed by PIPE‐T. (A) Box plots indicate the average number of detectable EV‐miRs in HYase‐treated EDTA (sample 1B) and heparin/de‐heparinized (sample 2D) SF samples. Boxes comprise the values falling between the 25th and 75th percentiles, median values are represented by horizontal lines, and the highest and lowest values for each group are represented by whiskers (lines that extend from the boxes). Statistical analysis using paired Student's *t*‐test was performed. *p* value of sample 2D relative to sample 1B: ***p < .001. (B) Heat‐map representation and unsupervised hierarchical clustering analysis of differentially expressed EV‐miRs in HYase‐treated EDTA and heparin/de‐heparinized groups of SF samples. EV‐miR expression levels were z‐scored and log2 transformed and are indicated by a two‐colour scale ranging from blue (lowest values) to red (highest values) reported in the horizontal bar at the top of the figure. Each column represents a patient (indicated on the top side) and each row represents an EV‐miR. Dendogram reports the results of the unsupervised hierarchical clustering and is displayed at the top of the plot. Two main clusters of patients were identified and displayed at the top of the plot. A good association between clusters and the EDTA and heparin/de‐heparinized groups of samples is shown. (C) Box plots show the mean RQ expression of nine representative differentially expressed EV‐miRs in SF samples from the eight patients analysed. Boxes comprise the values falling between the 25th and 75th percentiles, median values are represented by horizontal lines, and the highest and lowest values for each group are represented by whiskers (lines that extend from the boxes). Data were analysed with paired Student's *t*‐test and are expressed in log2. *p* value of sample 2D relative to 1B: **p < .01; ***p < .001.

To identify EV‐miRs that mostly contributed to OJIA joint pathogenesis, we conducted a comparative analysis of the miRNome of EVs isolated from SF with respect to PL samples (Figure S4) of newly diagnosed OJIA patients. We detected clearly distinct EV‐miR expression patterns (Figure 3A,B). Differential expression analysis identified 24 and 79 EV‐miRs significantly up‐ and downregulated, respectively, in SF versus PL (Table S4). EV‐miR‐regulated processes were defined by pathway analysis based on validated target genes of the differentially expressed EV‐miRs using MirWalk and GO/KEGG ontologies. Enriched processes/pathways were associated with each modulated EV‐miR and relative target genes using miRNet (Supporting Information, Section 2.10). This analysis revealed the significant enrichment of terms related to inflammation, cartilage/bone homeostasis, hypoxia, cell damage/death and hormone metabolism. A selection of the processes with the most significant enrichment score is reported in Table S5 (Supporting Information, Sections 3.4 and 3.5). We defined a subset of 15 EV‐miRs deregulated in SF versus PL targeting multiple genes critically involved in most of the enriched pathways (Figure 3C), among which the seven upregulated are endowed with pro‐inflammatory/pro‐apoptotic functions, whereas the eight downregulated are characterized by anti‐inflammatory/anti‐erosive activities^7^, ^10^ (Supporting Information, Section 4). Expression changes were confirmed by qRT‐PCR performed on selected EV‐miRs (Figure S5). Target genes of identified EV‐miRs mainly coded for cytokines/chemokines, signalling molecules, transcription factors, cell‐cycle/apoptosis regulators and stress factors (Tables S6 and S7). These findings suggest that alterations in the levels of specific EV‐miRs within OJIA joints may contribute to the promotion of synovitis and joint damage and the inhibition of processes involved in the resolution of inflammation (Supporting Information, Section 4).

**FIGURE 3:**
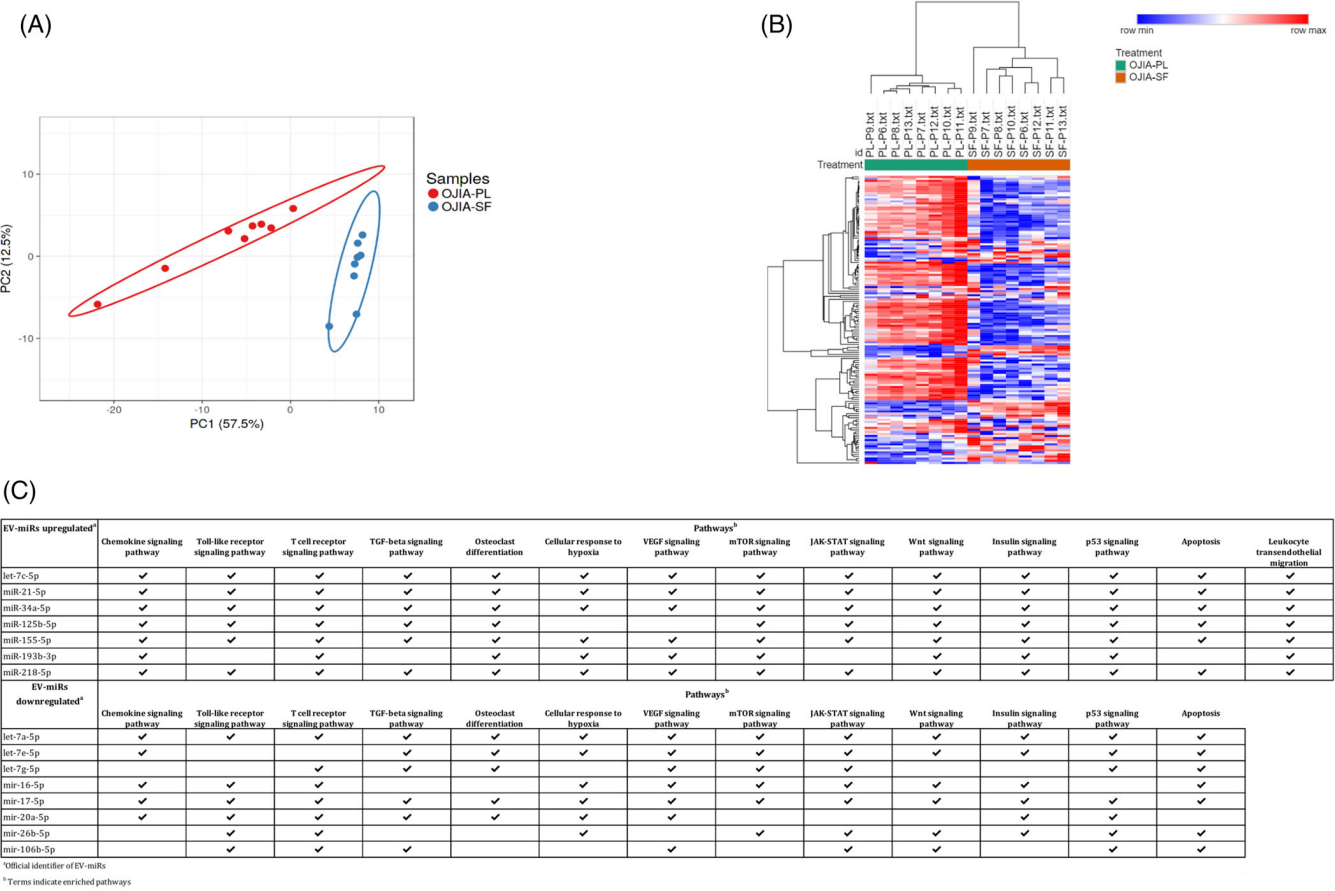
**Comparative analysis of EV‐miR expression profiles between paired SF and PL specimens from new‐onset OJIA patients**. EV‐miRs were profiled in paired SF and PL specimens obtained from patients of the validation cohort, and pathway analysis was carried out on EV‐miRs differentially expressed in SF versus PL based on experimentally validated miRNA‐targets. (A) Principal component analysis (PCA) of paired SF and PL samples based on differentially expressed EV‐miRs. The percentage of the total variation accounted for the first and second components is shown on the x and y axes, respectively. The ellipse around a group of samples indicates the estimated region where a new observation from that group would fall in with a confidence level of .95. Each point indicates a sample and is coloured in blue or red according to the sample type. (B) Heat‐map representation and unsupervised hierarchical clustering analysis of EV‐miR profiles in SF and PL from OJIA patients. EV‐miR expression levels are indicated as described in the legend in Figure 2B. Two patient clusters were identified and displayed on the top of the plot for the SF and PL samples. An optimal association between clusters and groups is shown. (C) List of the seven over‐expressed and eight under‐expressed EV‐miRs in SF and PL samples mostly represented in significantly enriched GO biological processes and KEGG pathways. Significantly enriched processes/pathways were associated with each of the EV‐miRs differentially expressed in SF versus paired PL samples by miRNet2.0 software. The selected EV‐miRs and associated pathways are indicated.

To identify EV‐miRs that could represent early putative diagnostic biomarkers, EV‐miR expression profiles were compared between new‐onset OJIA patients and age/gender‐matched CTR (Table S1, Supporting Information, Sections 2.1 and 3.6). OJIA‐SF and CTR‐PL samples clearly clustered in two groups based on their EV‐miRs expression levels (Figure 4A,B). A total of 54 EV‐miRs were identified, most of which were the same identified upon comparison with paired OJIA‐PL samples (Table S4), confirming the existence of an SF‐associated EV‐miR signature with value as a molecular indicator of the joint pathologic state. A marked separation between OJIA‐PL and CTR‐PL samples was also demonstrated (Figure 4C,D). We detected a panel of 110 circulating EV‐miRs differentiating OJIA patients from CTR among which 106 were significantly upregulated and 4 significantly downregulated in patient specimens (Table S8), suggesting specific disease‐related changes in the expression levels of circulating EV‐miRs. Interestingly, Venn diagrams showed consensual overexpression of a subset of 16 EV‐miRs in SF and PL from OJIA patients compared to CTR‐PL (Figure 4E), potentially representing a disease‐specific EV‐miR signature measurable at both local and systemic levels able to discriminate new‐onset OJIA patients from healthy children. Differential expression of selected EV‐miRs was validated by qRT‐PCR (Figure S6). The area under the ROC curves (AUC) generated for each of the 16 consensually upregulated EV‐miRs showed a clear visual discriminating power of their expression levels between OJIA and CTR groups (Figure 4F and Table S9), indicating a high potential diagnostic value (Supporting Information, Section 3.6).

**FIGURE 4:**
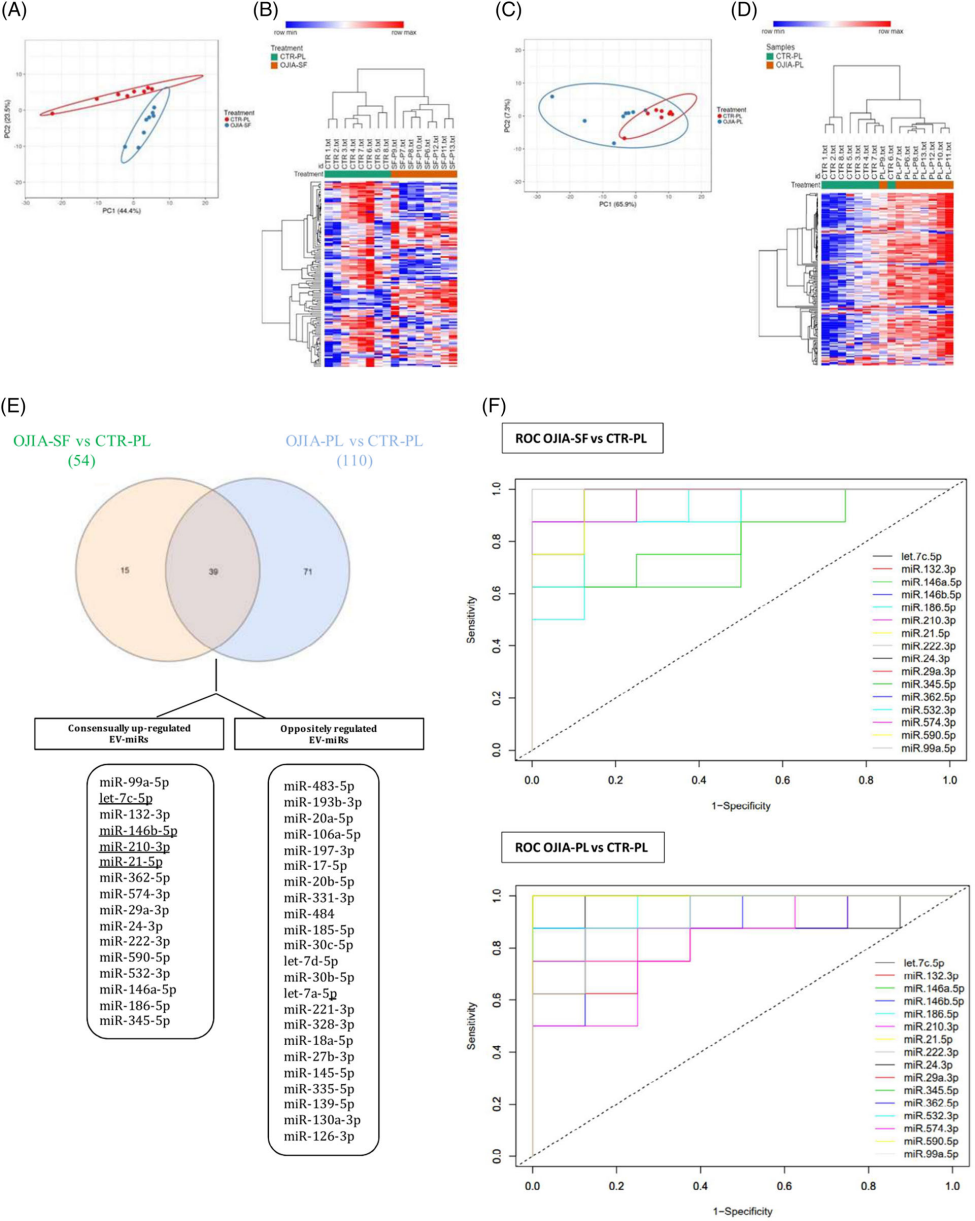
**Comparative analysis of EV‐miR expression profiles between new‐onset OJIA patients and control children**. EV‐miRs were profiled in paired OJIA‐SF and OJIA‐PL specimens obtained from the eight patients of the validation cohort and PL samples from healthy children (CTR‐PL). (A) PCA of EV‐miR expression profiles in OJIA‐SF and CTR‐PL specimens. Data are represented as detailed in the legend of Figure 3A. (B) Heat‐map representation and unsupervised hierarchical clustering analysis of differentially expressed EV‐miRs in OJIA‐SF and CTR‐PL samples, indicated as described in the legend of Figure 2B. Two patient clusters were identified by the unsupervised hierarchical method. An optimal association between the clusters and the OJIA‐SF and CTR‐PL samples is shown. (C) PCA of EV‐miR expression profiles in OJIA‐PL and CTR‐PL samples. Data are represented as detailed in the legend of Figure 3A. Each point indicates a sample and is coloured in blue or red according to the sample type. (D) Heat‐map representation and unsupervised hierarchical clustering analysis of significantly differentially expressed EV‐miRs in OJIA‐PL and CTR‐PL samples is indicated as described in the legend of Figure 2B. Two patient clusters were identified by the unsupervised hierarchical method. A good association between the clusters and the OJIA‐PL and CTR‐PL samples is shown. (E) Venn diagrams show the number of common and exclusive EV‐miRs detectable in OJIA‐SF and OJIA‐PL versus CTR‐PL specimens. Both up‐ and downregulated EV‐miRs were considered. Common EV‐miRs displaying consensual and opposite modulation in paired OJIA‐SF and OJIA‐PL samples with respect to CTR PL specimens are indicated. Underlined miRNAs have been validated by qRT‐PCR. (F) ROC curves of consensually upregulated EV‐miRs between OJIA‐SF and CTR‐PL or OJIA‐PL and CTR‐PL are displayed on the top or bottom part of the plot, respectively. ROC curves were generated by the easyROC tool. The x‐axis reports the 1‐specificity values. The y‐axis reports the sensitivity values. Distinct colours were used to separate curves from different EV‐miRs. The legend is reported in the bottom right section of each plot. The dashed line indicates the performance of the random classifier.

In conclusion, this study represents the first detailed characterization of the miRNome of EVs isolated from new‐onset OJIA patients, instrumental for a better understanding of disease molecular mechanisms. Our results define EV‐miR signatures potentially implicated in OJIA pathogenesis and that could represent new early molecular indicators of OJIA development and/or candidate biomarkers for the refinement of patient diagnosis. These data also provide the bases for investigating the potential of EV‐miRs as novel targets for tailored treatment strategies in OJIA, fostering the implementation of personalized therapeutic interventions. A prospective study is currently ongoing to validate these results in a larger cohort of patients and determine EV‐miR signature specificity for the OJIA subtype and correlation with patient clinical parameters for the prediction of disease outcome.

## CONFLICT OF INTEREST

The authors declare no conflict of interest.

## Supporting information

Supplementary information

Supplementary information

Supplementary information
