# Supplementary material for: Extracellular vesicle‐derived microRNAs as potential biomarkers in oligoarticular juvenile idiopathic arthritis patients: methodological challenges and new perspectives
Source: Clin Transl Med. 2022 Sep 30;12(10):e1067. doi: 10.1002/ctm2.1067 (PMC9523680; doi:10.1002/ctm2.1067)
Supplement: Supplementary file 1 — Supplementary information [file CTM2-12-e1067-s001.docx]

**Supporting Information**

**Extracellular vesicle-derived microRNAs as potential biomarkers in Oligoarticular Juvenile Idiopathic Arthritis patients: methodological challenges and new perspectives**

[1](#_Toc112834613) BACKGROUND 2

[2 MATERIALS AND METHODS 4](#_Toc112834614)

[2.1 Study population 4](#_Toc112834615)

[2.2 Sample collection 5](#_Toc112834616)

[2.3 EV isolation 5](#_Toc112834617)

[2.4 Dynamic Light Scattering (DLS) analysis of EVs 6](#_Toc112834618)

[2.5 Transmission electron microscopy (TEM) analysis 6](#_Toc112834620)

[2.6](#_Toc112834621) Nanoparticle Tracking Analysis (NTA) 6

[2.7 Western Blot (WB) analysis 7](#_Toc112834622)

[2.8 EV-RNA extraction 7](#_Toc112834623)

[2.9 EV-miR expression profiling by Quantitative Real Time PCR (qRT-PCR) 8](#_Toc112834624)

[2.10 Statistical analysis 8](#_Toc112834625)

[3 RESULTS 11](#_Toc112834626)

[3.1 Characterization of EVs isolated from the SF of new-onset OJIA patients 11](#_Toc112834627)

[3.2 Evaluation of miRNA content in SF-derived EVs 12](#_Toc112834628)

[3.3 Analysis of EV-miR expression profiles in SF samples from new-onset OJIA patients 13](#_Toc112834629)

[3.4 EV-miR expression patterns differ between paired SF and PL samples from new-onset OJIA patients 14](#_Toc112834630)

[3.5 Pathway analysis of differentially expressed EV-miRs reveals the enrichment of target genes associated with inflammation, cartilage/bone homeostasis, hypoxia, and hormone metabolism 15](#_Toc112834631)

[3.6 EV-miR expression profiles differentiate new-onset OJIA patients from control children 17](#_Toc112834632)

[4 DISCUSSION 18](#_Toc112834633)

[5 REFERENCES 26](#_Toc112834634)

# BACKGROUND

OJIA is a chronic inflammatory arthritis (prevalence 1-5/10000) whose causes remain mostly unknown, which arises in four or fewer joints of genetically susceptible children after exposure to infective/traumatic triggers or surgery and is characterized by early onset (≤6 years of age), female predominance, asymmetry, high frequency of anti-nuclear antibody (ANA), strong association with HLA-DRB1*0801, and elevated risk of chronic uveitis^1-3^. Considerable heterogeneity in disease clinical course and outcome exists. Despite significant therapeutic advances were obtained in the last decades improving patient management, a high proportion (up to 40%) of children affected by OJIA does not respond adequately to current medications or fails to achieve sustained clinical drug-free remission, showing recurrence in treated joints and/or progressive spread to other joints, and carrying an increased risk of cartilage and bone erosion, which may lead to structural joint damage and functional impairment^4, 5^. Early diagnosis and prediction of disease evolution over time and response to treatment in individual patients are crucial for setting up more tailored therapies but are difficult due to the lack of validated biomarkers^6, 7^. To date, only a few immune indicators have in fact been proposed as potential biomarkers for OJIA^8, 9^. Therefore, a great deal of efforts is currently being expended in the search of new non-invasive biomarkers readily measurable in patient-derived material at an early stage of the disease and in a minimally invasive way^7, 10, 11^. Incorporation of effective and reliable biomarkers into routine practice could facilitate appropriate disease management, fostering the implementation of personalized and targeted therapeutic interventions. A better understanding of the molecular regulatory mechanisms underlying OJIA pathophysiology is essential for identifying new early biomarkers and molecular targets for tailored therapies

Recently, a role for extracellular vesicles (EVs) in the pathogenesis of many diseases^12, 13^, including those caused by dysfunctions of the immune system^14-16^, and potential as a source of biomarkers and therapeutic targets in these diseases were suggested. EVs are a heterogeneous group of lipid bilayer membrane-delimited nanometer-sized vesicles actively released by most cell types into biological fluids, which act as important mediators of intercellular communication, exerting key functions in immune surveillance, cell proliferation, differentiation, and apoptosis^17, 18^, They have been traditionally classified into two major subgroups based on differences in biogenesis and size, namely exosomes (30-150 nm in diameter), which originate by fusion of endosomal multivesicular bodies with the plasma membrane, and microvesicles (100-1000 nm in diameter), formed by outward budding of the plasma membrane, although overlap between these two types of vesicles was largely reported^19, 20^. Important EV constituents that may fulfill biomarker requirements are represented by miRNAs^21, 22^, short (≈20-22 nt) non-coding single-stranded RNAs functioning as gene negative post-transcriptional regulators and involved in the epigenetic control of multiple processes, including inflammatory responses and immune cell differentiation, maturation, and functions^23-25^. Altered miRNA profiles are closely linked to the development and progression of various chronic inflammatory and autoimmune disorders^26^ and have been suggested to have potential diagnostic/prognostic value in patients with different types of rheumatic diseases^27-29^, including systemic and polyarticular JIA^30-32^.

A great research effort has been recently focused on the analysis of EV-miRs in blood and other biological fluids to identify new predictors of disease development/activity and define patient stratification and treatment response in several clinical settings^11, 21^, including in patients affected by adult arthritides^15, 33^. Analysis of EV-associated miRNAs present several advantages respect to that of “free circulating” miRNAs for new biomarker discovery. Firstly, intravesicular miRNAs are highly protected from endogenous enzymatic degradation and stable even under suboptimal storage conditions and in the presence of RNAse, providing an enriched and consistent source of biomarkers^34^. On the contrary, “free-circulating” miRNAs are more sensitive to pre-clinical variables of sample handling (time of processing, storage conditions) and at an increased risk of degradation by RNAse present in biological fluids, which could significantly decrease their concentration and alter their integrity upon collection, being thus more prone to variations in expression levels^21, 35^. EV isolation could, thus, improve the detectability of low-abundance miRNAs, which could otherwise be missed by the analysis of unfractionated samples, reducing the probability of false negative results, and allow to exclude damaged molecules^21^. Secondly, EVs contain a tissue-type miRNA signature. Because the loading of EV cargo is an active and strictly regulated mechanism, its composition will provide better reflection of the status of its producing cell compared to “free” molecules, and analysis of miRNA enclosed in EVs may thus improve their specificity for the pathologic conditions. EV-miR profile was in fact reported to vary as a function of the pathological situation and to be representative of the parental diseased cells ^36, 37^. Thirdly, the load of EVs released in the pathologic state is significantly increased respect to the correspondent normal tissue, as evidenced by their higher concentrations in biofluids, resulting in the enrichment of miRNAs biomarkers^37^ Finally, an appealing feature of EV-based biomarker analysis is the significant reduction in the sample complexity compared to whole bodily fluids, which is another rate-limiting factor of biomarker analysis. These characteristics increase assay sensitivity and specificity and make EV-miR analysis highly instrumental for new biomarker discovery. Generation of several putative EV-miR markers have, in fact, been reported in various clinical settings^37^

No information is currently available on EV-miR-associated biomarkers in OJIA, Because joints are the main targets of disease clinical manifestations, it is probable that the most relevant biomarkers for this disease would be localized within affected joints^38, 39^. Synovial fluid (SF) reflects the biological milieu of the joint and may, thus, offer a direct measure of its pathologic state^28^. Previous studies in patients affected by adult rheumatic arthritis have shown that EVs are actively released into the SF by local and infiltrating immune cells and contribute to the perpetuation of joint inflammation, synovial cell proliferation, and cartilage degradation^16, 38^. The characterization of miRNA carried out by EVs in SF samples of new-onset OJIA patients could, thus, provide a powerful mean to elucidate the molecular pathogenetic mechanisms of OJIA development and yield novel early candidate miRNA-based biomarkers for clinical use and potential therapeutic targets^15, 33^.

# MATERIALS AND METHODS

## Study population

Thirteen patients newly diagnosed with OJIA at the Pediatric and Rheumatologic Department of the IRCCS Gaslini Institute, Genova, Italy on the basis of the 2001 International League of Associations for Rheumatology (ILAR) classification criteria^40^ were enrolled consecutively in the study from October 2018 through February 2020 (Table S1). All patients had clinically active disease, with joint effusion, swelling, pain, and stiffness, and underwent arthrocentesis and intra-articular steroid administration as part of routine clinical care. Arthrocentesis was performed with or without ultrasound guidance under local anesthesia or, in case of younger patients or multiple joints, under general anesthesia. Patients receiving systemic treatment (steroids, disease-modifying anti-rheumatic drugs-DMARDs, or biological therapy) at the time of arthrocentesis were excluded from the study. Eight age- and gender-matched children undergoing minor orthopedic procedures at the Gaslini Institute were enrolled as control group. Detailed clinical and laboratory examination of control subjects was carried out to rule out infections, inflammatory, and chronic diseases. Age and sex (male vs female) were tested as potential confounding factors between CTR subjects and OJIA patients by performing a Student’s t test or a Fisher’s exact test, respectively. P value less than 0.05 was considered statistically significant. The protocol of the study was reviewed and approved by the Ethics Committee of the Liguria Region (Approval 165/2018) and authorized by the General Director of the Gaslini Institute, and the procedures were carried out according to the approved guidelines and in adherence to the general ethical principles set forth in the Declaration of Helsinki. Written informed consent to participate to the study was obtained from the parents or the patient legal guardian prior to sample collection.

## Sample collection

Synovial fluid (SF) aspirates were collected from the joints of OJIA patients by arthrocentesis under vacuum and split into tubes containing EDTA or sodium-heparin, according to the flow chart depicted in Figure S1. Paired peripheral blood (PB) samples and PB from control children were obtained by venipuncture and collected in EDTA tubes. Specimens were centrifuged at 500 x g for 10 minutes at room temperature (RT) within 2 hr of collection to obtain cell-free SF and plasma (PL) and stored at -80°C until use.

## EV isolation

EV isolation from 500 μl of SF and PL samples was performed using the exoRNeasy Serum/Plasma Midi kit (Qiagen Italia, Milano, Italy), that uses membrane affinity spin columns to efficiently capture intact EVs from small sample volumes^41^, according to the manufacturer instructions. Cell-free SF samples were either treated with 2U/ml Hyaluronidase (HYase) (Sigma, Merck Life Science, Milano, Italy) for 30 minutes at 37 ^0^C to remove contaminating hyaluronan extracellular matrix (ECM) components or left untreated prior to EV isolation. 500 μl SF samples were firstly centrifuged at 500 x g for 10 minutes at RT and then at 16,000 ×*g* for 15 minutes at RT to eliminate cellular debris. PL samples, not undergoing HYase treatment, were directly centrifuged at 16,000 ×*g* for 15 minutes. Supernatants were mixed with one volume of XBP binding buffer and loaded onto exoEasy spin column to bind EVs to the membrane. After centrifugation at 500 x g for 1 minute at RT, the flow-through was discarded and the column was washed with 3.5 ml of XWP Washing Buffer and spun at 5,000 x g for 5 minutes at RT, to wash-off not specifically bound material. The spin column was transferred to a new collection tube, and intact EVs were eluted by addition to the column of 100 μl of Buffer XE. EV quantification was obtained by surface protein count using the standard Bradford protein assay (Bio-Rad, Milano, Italy).

## Dynamic Light Scattering (DLS) analysis of EVs

Isolated vesicles were characterized in terms of mean size distribution by DLS using the Zetasizer Nano ZS90 particle size analyzer (Malvern Instruments, Worcestershire, UK), as detailed ^42^. Light scattering from the samples was detected by a photomultiplier tube placed at a 90° fixed angle to the incident laser beam and at a constant temperature of 25°C. Nanosphere™ size standards with a mean diameter of 57 ± 4 nm (Thermo Scientific) were used to verify the correct operation of the particle size analyzer The homogeneity of vesicles size distribution was determined by the polidispersity index (PDI)^43^. The analysis was replicated on different samples.

## Transmission electron microscopy (TEM) analysis

Ultrastructural analysis of EVs preparations isolated from SF and PL samples was performed by TEM as described^44^. Briefly, EVs were resuspended in 20 μL PBS (pH 7.4) and fixed by adding an equal volume of 2% paraformaldehyde in 0.1 mol/L phosphate buffer (pH 7.4). EVs were then adsorbed for 10 minutes to formvar-carbon coated copper grids by floating the grids on 5 μl drops on parafilm. Subsequently, grids with adhered EVs were rinsed in PBS and negatively stained with 2% uranyl acetate for 5 minutes at room temperature. Stained grids were embedded in 2.5% methylcellulose for improved preservation and air dried before examination. Electron micrographs were taken at Hitachi TEM microscope (HT7800 series, Tokyo, Japan) equipped with Megaview 3 digital camera and Radius software (EMSIS, Germany).

## Nanoparticle Tracking Analysis (NTA)

EV **sizes** were measured by NTA using a Nanosight NS300 (Malvern), equipped with a 352 nm green laser and a high sensitivity sCMOS camera, as described ^45^. Briefly, samples were diluted in 1 ml of particle-free PBS to reach the ideal measurement concentration within a range of 20-100 particles per frame, based on preliminary tests, according to the manufacturer’s recommendations . Specifically, SF and PL samples had to be diluted 1:500 and 1:1000, respectively. Samples were manually injected in the sample chamber with sterile 1-ml syringes. Camera level was increased until all particles were distinctly visible not exceeding a particle signal saturation over 20%, and the ideal detection threshold was determined by limiting the blue crossing to 4 per frame. Each sample was measured 5 times under constant flow conditions (flow rate of 50 µL/sec) and a temperature of 25 °C. For each measurement five 1-min videos of 45s each were captured at a frame rate of 25 frames/second. In order to minimize data skewing based on single large particles, the number of completed tracks in NTA measurements was always above the minimum value of 1000. Individual particles were tracked. Data have been analyzed by the in-built NanoSight Software v3.4.4 and the final histogram of each sample is the result of an average of the multigraph of the five analyzes performed.

## Western Blot (WB) analysis

Expression of the EV tetraspanins, CD9, CD63, and CD81 was assessed by WB analysis. Briefly, EVs were lysed with the RIPA Buffer (Thermo Fisher Scientific, Milano, Italy) containing a protease inhibitor cocktail (Roche SpA, Monza, Italia), and whole protein extracts (2-10 μg according to the Bradford method) were resolved on a precast 4-12% Tris-Glycine Mini Gel (Thermo Fisher Scientific) and transferred to polyvinylidene fluoride (PVDF) membranes (Thermo Fisher Scientific). The blot was hybridized overnight at 4°C with primary mouse mAbs specific for CD9 (size 24 kDa) (Santa Cruz Biotechnology, Heidelberg, Germany), CD63 (size range 30-60 kDa) (EXOAB-KIT-1, System Bioscences, CA, USA), or CD81 (size 26 kDa (EXOAB-KIT-1 System Biosciences)^46-48^, followed by a horseradish peroxidase (HRP)-conjugated secondary goat anti-mouse Ab (Thermo Fischer Scientific), incubated for 1 h at room temperature. Chemiluminescence detection was carried out using an ECL Select kit (Sigma), according to the manufacturer's instructions. Imaging was performed with a ChemiDoc Touch Imaging System (Bio-Rad Laboratories).

## EV-RNA extraction

To purify small-RNAs from EVs, the exoRNeasy serum/plasma kit was combined with QIAzol. The elution step was substituted with the lysis step through addition of 700 μl of QIAzol to the membrane of the exoRNeasy spin column and centrifugation at 5,000 x g for 5 minutes at RT. Total RNA extraction was then performed according to the manufacturer’s instructions. In some samples collected in sodium-heparin tubes, the RNA preparations were treated with 1U heparinase (Sigma) I/μg RNA at 25°C for 1h prior to reverse transcription, to remove heparin, as previously described^49^. Quality control and quantitative assessment were carried out by capillary electrophoresis with an Agilent 2100 Bioanalyzer, using the Small RNA Assay (Agilent Technologies Spa, Milano, Italy).

## EV-miR expression profiling by Quantitative Real Time PCR (qRT-PCR)

EV-miR expression profile was analyzed by the TaqMan Array Card Technology. Briefly, 50 ng of EV-RNA were reverse transcribed into double-stranded cDNA on a GeneAmp PCR System 2700 thermal cycler (Applied Biosystems, Milano) with the TaqMan® microRNA Reverse Transcription Kit, using the MegaplexTM RT Human Pool A (Thermo Fisher Scientific). cDNA was amplified by TaqMan®PreAmp Master Mix using MegaplexTM Pre-Amp primers Human Pool A. The pre-amplification product was used to perform miRNA expression profiling using the TaqMan-Array Human MicroRNA A Cards (Thermo Fisher Scientific), which contain a total of 381 human targets, run on the ViiA7 Real Time PCR System (Thermo Fisher Scientific), as detailed previously^50^. Selected EV-miRs (let-7c, miR-21-5p, let-7a, miR-16-5p, let-7g, miR-210-3p, miR-146b-5p) were validated by qRT-PCR using specific TaqMan miRNAs Assays (Thermo Fisher Scientific). Samples were run in triplicate on MicroAmp Fast Optical 96-well reaction plate (Thermo Fisher Scientific, Monza, MB, Italy). CT observed by qRT-PCR were compared with those obtained with the array cards to confirm differential expression of specific EV-miRs in the distinct groups of samples analyzed. Statistical analysis and plots were carried out using GraphPad Prism 6.0 for Windows (www.graphpad.com).

## Statistical analysis

Data processing, categorization, normalization, filtering, imputation, and differential expression was carried out by PIPE-T, a new Galaxy tool developed in our laboratory that integrates the functions implemented in several open-source R packages into one reusable, transparent, and accessible wrapper^51^. Raw data were labeled on the basis of 14-32 threshold cycle (CT) expression range and quality flag values. CT values falling within the range of 14-32 were considered as reliable, whereas those higher than 32, lower than 14, or undergoing an experiment failure were categorized as undetermined (NA), according to the manufacturer guidelines. Reliable data were normalized using the Global Mean method to reduce technical variability among samples^51^. Briefly, the global mean value of each sample was calculated by subtracting the arithmetic mean of the CT values from the raw CT of each EV-miRs (ΔCT). As NA can adversely affect downstream analysis^52^, we set up a filtering threshold of at most 20% NAs. Only EV-miRs with ≤20% of NA were retained for the analysis. The NA of the EV-miRs that passed the filter were imputed using the Mestdagh imputation method^53^ to be processed in subsequent analysis steps. The method consists in substituting each NA of a EV-miR with a numeric value estimated as the lowest EV-miR expression value subtracted of an additional log2 unit^51^. The Student’s t test method was used to identify significant differentially expressed EV-miRs. The significance of EV-miR expression differences between samples derived from the same patient was calculated using the paired Student’s t-test, whereas the unpaired Student’s t test was used to identify differences in the EV-miR expression levels when the comparison involved samples derived from patients and control subjects. The p value was adjusted for multiple hypothesis testing by the Benjamini-Hochberg method. Fold-change (FC) was calculated to determine the magnitude of the EV-miR expression differences. EV-miRs exhibiting an adjusted p value ≤ 0.05 and FC ≥ 2 or ≤ 0.5 were considered significantly differentially expressed. We referred to –ΔCT as RQ value. The full set of data has been deposited at the Gene Expression Omnibus (GEO) public repository at NCBI (www.ncbi.nlm.nih.gov) and can be accessed to through GEO Series accession number (GSE191326).

Heat map representation and unsupervised hierarchical clustering analysis were carried out to visualize the expression of differentially modulated EV-miRs using Morpheus versatile matrix visualization and analysis software (Morpheus, Broad Institute, Cambridge Massachusetts, USA). Principal Component Analysis (PCA) was performed to emphasize the differences among samples using ClustVis software^54^. Overlapping and exclusive elements among the lists of Ev-miRs were defined by Venn diagrams^55^. The significance of the overlapping was estimated with hypergeometric statistics using the Stats R package (http://www.R-project.org/). Pathway analysis was carried out for validated EV-miR target genes using MirWalk 3.0^56^ and the gene ontology (GO) or Kyoto Encyclopedia of Genes and Genomes (KEGG) gene set collections. Terms or pathways with an adjusted p-value ≤0.05 were considered significantly enriched. The miRNet 2.0 web-based platform^57^ was used to associate each significantly enriched process/pathway identified by miRWalk with the specific EV-miRs and relative target genes. Receiver operating characteristic (ROCs) curves were plotted to display the discriminating power of each significantly modulated EV-miR across groups. Area under the ROC curve (AUC) was computed by the easyROC tool^58^ to quantitively assess the discriminating performance of each EV-miR. EV-miR expression was used to generate ROC curves.

# RESULTS

## Characterization of EVs isolated from the SF of new-onset OJIA patients

Experiments were carried out to characterize EVs released into the SF of OJIA patients. SF samples were collected from thirteen new-onset OJIA patients with clinically active disease at the time of therapeutic arthrocentesis. Patients were grouped into two independent cohorts: a training (n=5, P1-P5) and a validation (n=8, P6-13) cohort. Patient clinical features, various laboratory parameters, and known markers of disease activity are reported in Table S1.

The use of anticoagulants is necessary for the study of SF because of frequent blood contamination, but divergent information are available on which type of anticoagulant should be used^59-61^. In addition, the presence in SF of elevated concentrations of high-molecular-weight hyaluronic acid (HA) complexed with synovial proteins^62^ represents a challenge for EV-miR analysis, because it increases fluid viscosity and EV trapping with consequent reduction of EV-miR purification efficiency and quality^63, 64^. The effects on EV isolation of two anticoagulants commonly used for SF sample collection, EDTA and sodium-heparin, were compared. Some specimens were treated with HYase to evaluate whether EV isolation would benefit from the digestion of HA contaminants. A flow chart summarizing the main steps of sample processing and analysis is shown in Figure S1.

EVs were purified from 500 μl SF samples and characterized in terms of recovery, dispersion, size, and morphology and for the expression of the typical EV markers, CD9, CD63, and CD81 ^65, 66^. EV recovery was measured as μg of total protein count normalized to 1 ml of sample. As reported in Figure 1a, mean protein concentrations were comparable in specimens collected in EDTA (53.5 μg/ml) and sodium-heparin (59.5 μg/ml). Pretreatment with HYase markedly reduced SF viscosity and facilitated sample elution through the column, significantly increasing EV yield in both EDTA (mean protein 87.1 μg/ml) and heparin (mean protein 79.5 μg/ml) samples. Similar EV characteristics were observed in all tested samples, regardless of the anticoagulant used. DLS revealed a bell-shaped size distribution profile of EVs that peaked between 345 and 380 nm with an average PDI of 0.183, indicative of relatively monodisperse nanometer-sized populations (Fig.1b). TEM allowed to clearly visualize the isolated vesicles surrounded by the lipid bilayer (Fig.1c). EVs appeared as round- or vesicles, some of which presenting internal membranes ^67^, with size ranging between 51 and 354 nm consistent with the presence of a mixed population of both exosomes and microvesicles, although the majority had a diameter smaller than 120 nm. Low degree of clustering (cluster size 500-600 nm) was also detected (data not shown). NTA confirmed enrichment for particles smaller than 300 nm, with an average modal and mean diameter ranging from 156.1 to 240,3 and from 180.1 to 196,4, respectively, in the three samples analyzed and ≤10% of particles with a diameter exceeding 300 nm, with some patient-to-patient variation (Fig.1d). WB analysis demonstrated that isolated vesicles expressed high levels of CD9 and CD63, whereas CD81 was not detected (Fig.1e).

These data suggest that EV isolation from SF samples is not substantially influenced by the type of anticoagulant used but is improved by the removal of contaminating HA.

## Evaluation of miRNA content in SF-derived EVs

The impact of anticoagulants and HYase pre-treatment on miRNA isolation and detection was then assessed. Total RNA was extracted from SF-derived EVs, and the miRNA content was evaluated by capillary electrophoresis using the small RNA Assay. As depicted in Figure 1f, small-RNA patterns differed extensively in the electropherograms of samples collected in EDTA (±HYase) respect to those in sodium-heparin (±HYase) tubes. EV purified from EDTA samples showed a narrow-size distribution pattern typical of small-RNAs and contained a significant RNA fraction in the region between 6 and 40 nucleotides in length, consistent with miRNA size (samples 1A,1B). In contrast, samples collected into sodium heparin showed a non-conventional small-RNA profile characterized by higher and larger peaks, suggesting that heparin impairs small-RNA detection (samples 2A,2B). Heparin removal by RNA treatment with heparinase I prior to reverse transcription restored a more typical pattern of small-RNAs, in particular in samples pretreated with HYase (samples 2C,2D).

Total RNA was then reverse transcribed, pre-amplified, and analyzed by TaqMan-Array-Human MicroRNA A Cards, that allowed the measurement of 381 miRNAs for each sample through qRT-PCR. Samples collected in EDTA displayed reliable amplification curves, and HYase pre-treatment led to higher EV-miR amplification, as indicated by lower CT values (Fig.1f, samples 1A,1B). In contrast, no RNA amplification was observed in samples collected into sodium-heparin tubes, independently from HYase treatment (Fig.1f, samples 2A,2B). Heparin inhibitory effects were reversed by RNA pre-treatment with heparinase I, which enabled RNA amplification (Fig.1f, samples 2C,2D) and was, thus, used in all the following analyses.

These data indicate that the choice of anticoagulant for SF sample collection can affect EV-miR detection and that de-heparinization is required when sodium-heparin is used.

## Analysis of EV-miR expression profiles in SF samples from new-onset OJIA patients

To evaluate the performance of the different sample processing procedures on EV-miR expression profiles, raw qRT-PCR expression data were analyzed using PIPE-T^51^. We first examined SF samples from patients of the training cohort. As depicted in Figure S2a, the number of detected EV-miRs (CT 14-32) was higher in EDTA (sample 1A, mean±SD: 122±26.6) than in heparin (sample 2C, 77.4±38.4) and was further increased (142±29.8) upon HYase treatment, which in contrast appeared to decrease EV-miR detection in samples collected in heparin (52.4±28) (p≤ 0.01, samples 1B vs 2D). Both shared and exclusive EV-miRs were observed by Venn diagrams in the different samples (Fig.S2b). A core set of EV-miRs (average number 52, range 35-63 in the five patients) was common to all the groups analyzed. In addition, a subset of EV-miRs was shared exclusively by samples collected in EDTA treated with HYase (1B) or left untreated (1A) (average number 52, range 25-119), whereas almost no EV-miR was detected uniquely in the heparin/de-heparinized (±HYase) samples (2C,2D) (average number 0.25, range 0-1), suggesting that the efficiency of EV-miR detection is lower upon sample collection in heparin than in EDTA despite the de-heparinization procedure.

The levels of expression of common EV-miRs were then compared among the different groups of specimens, by applying a filter based on NAs (as detailed in the Materials and Methods). EV-miR expression differences were visualized by heat map and unsupervised hierarchical clustering analysis, which revealed a clearly distinct profile between the EDTA (±HYase) and the heparin/de-heparinized (±HYase) specimens, with higher EV-miR levels observed in the former (Fig.S3a, samples 1A; 1B) than in the latter (Fig.S3a, samples 2C; 2D). Our analysis identified a total of 33 EV-miRs whose expression levels were significantly higher in the EDTA (±HYase) respect to the heparin/de-heparinized (±HYase) samples (Table S2), several of which have been previously reported to play an important role in inflammatory and immune responses and in the pathogenesis of arthritides and considered as promising biomarkers for these diseases^27, 28, 68^. As shown in Figure S3b, which depicts the mean RQ expression values of nine representative miRNAs, expression in EDTA samples was increased by treatment with HYase (p≤ 0.05, samples 1B vs 2D), suggesting that HA removal is beneficial for EV-miR profiling.

To confirm these results in an independent set of samples, we profiled EV-miRs in patients of the validation cohort. As reported in Figure 2a, a significantly higher number of EV-miRs (p≤ 0.001) was detectable in the EDTA+HYase (sample 1B) respect to the heparin/de-heparinized+HYase (sample 2D) SF (mean number±SD: 163±17 *vs* 43±11). Accordingly, a clear separation between the two groups was observed on the basis of the EV-miR expression levels (Fig.2b) (with the only exception of p11), with increased expression detected in EDTA+HYase. We identified a total of 24 significantly upregulated EV-miRs, 16 of which were the same overexpressed in EDTA samples from the training cohort (Table S3, Fig.2c).

Taken together, these results highlight the requirement of EDTA combined with HYase for optimal miRNA profiling in EVs isolated from SF.

## EV-miR expression patterns differ between paired SF and PL samples from new-onset OJIA patients

Having established a reliable procedure for EV-miR profiling in SF, we searched for EV-miRs potentially implicated in joint pathogenesis by comparing the EV-miR expression profile of SF respect to that of paired PL samples from new-onset OJIA patients. EVs were purified from the PL (500 μl) of patients of the validation cohort and analyzed as described for SF samples. In all the PL samples analyzed by DLS, we observed the presence of a monodisperse vesicle population (average PDI 0.154) peaking between 209 and 245 nm (Fig.S4a). At the ultrastructural level, EVs showed the typical round or appearance with size comprised between 64 and 368 nm, although the majority was characterized by a diameter comprised between 106 and 168 nm (Fig.S4b). A few aggregates were also observed (data not shown). NTA confirmed enrichment for particles smaller than 200 nm, showing an average modal and mean diameters ranging from 113.2 to 181 and from 126.6 to 154.1, respectively, in the three samples analyzed and demonstrated the presence of fewer than 10% of vesicles larger than 200 nm, with some variation among different samples (FigS4c). WB analysis showed high expression levels of the CD9 and CD63 markers on isolated EVs, whereas CD81 was not detectable (Fig.S4d). EV recovery was comparable to that observed in SF samples collected in EDTA+HYase (mean protein concentration in 3 different samples: 90±7.5 μg/ml). A significant fraction of miRNAs was detected by capillary electrophoresis in the small-RNA profile from PL-derived EVs, and efficient qRT-PCR amplification was demonstrated (Fig.S4e). An average number of 222 EV-miRs out of 381 measured by the Array MicroRNA Cards was detected in the PL samples analyzed.

PCA and hierarchical clustering analysis were used to define SF and PL differences in EV-miR expression levels. As shown by the PCA reported in Figure 3a, SF and PL samples could be clearly clustered into two well-defined groups on the basis of their EV-miR expression profiles, with substantial homogeneity among samples belonging to the same group. Unsupervised hierarchical clustering analysis confirmed the presence of distinct EV-miR patterns (Fig.3b). A total of 103 differentially expressed EV-miRs were identified, among which 24 were up-regulated and 79 were down-regulated in SF respect to paired PL (Table S4).

These results indicate that the miRNA expression profile of EVs isolated from the joints of new-onset OJIA patients strongly differs from the systemic profile, thereby representing a molecular indicator of the pathologic state of the tissue.

## Pathway analysis of differentially expressed EV-miRs reveals the enrichment of target genes associated with inflammation, cartilage/bone homeostasis, hypoxia, and hormone metabolism

Understanding the biological processes in which EV-miRs are implicated may help to elucidate disease molecular mechanisms and be instrumental for the identification of new putative biomarkers and therapeutic targets. To define EV-miR-regulated processes in OJIA joints, we conducted a pathway analysis based on the target genes of the 103 EV-miRs (23 up- and 79 down-regulated) differentially expressed in SF *vs* PL samples using the MirWalk software and GO/KEGG ontologies. miRWalk allows to define EV-miR-targeted pathways based on predicted and/or validated miRNA-target interactions^56^. Given to the high false discovery rate of miRNA-target prediction algorithms (data not shown), we focused only on experimentally validated EV-miR targets. An enrichment score with adjusted p values ≤0.05 was considered significant. MirWalk identified 3249 validated target genes. MirWalk pathway analysis of EV-miR target genes showed the significant enrichment of 197 GO biological processes (122 associated to up- and 75 to down-regulated EV-miRs) and of 208 KEGG pathways (107 relative to up- and 101 to down-regulated EV-miRs) in SF respect to PL samples. A selection of the processes with the most significant enrichment score is reported in Table S5.

A significant proportion of EV-miR target genes was implicated in biological processes involved in inflammation and immune responses, such as TNF, NF-κB, cytokine-mediated, chemokine, TLR, and T cell receptor signaling pathways, and leukocyte transendothelial migration, or related to the maintenance of cartilage and bone homeostasis, such as TGF-β, TGF-β receptor, and FGF receptor signaling pathways, osteoclast differentiation pathways, autophagy, and cellular response to TGFβ stimulus, whose disruption plays a pivotal role in arthritis development and progression^69-71^. Pathway analysis of EV-miR targets also revealed the significant enrichment of terms related to hypoxia, a common feature of the inflamed rheumatoid synovium^3, 72^, including cellular response to hypoxia, HIF-1, VEGF, and VEGF receptor signaling pathways. PI3K/Akt/mTOR, JAK-STAT, WNT, MAPK, and FoxO signaling pathways. which are essential for the normal metabolism of joint tissues but may contribute to the pathogenesis and progression of arthritis when deregulated^73-76^, were also significantly enriched. A significant number of EV-miR-target genes was involved in cell damage- and death-related processes, such as p53 signaling pathway, apoptosis, cellular response to DNA damage and reactive oxygen species, which are relevant for both cellular homeostasis and inflammatory responses and linked to chronic inflammatory autoimmune conditions^77, 78^. Finally, we observed the significant enrichment of processes involved in the metabolism and signaling of estrogens and androgen receptor, prolactin, insulin/receptor, parathyroid and thyroid hormones, which have known regulatory effects on inflammatory/immune processes in several tissues^79-81^, suggesting a role of EV-miRs in the control of hormone profiles in the OJIA joint.

An independent computation approach was then carried out using the miRNet tool to specifically associate significantly enriched processes/pathways with each of the 103 differentially expressed EV-miRs and relative target genes. As indicated in Figure 3c, we could define a restricted subset of 15 EV-miRs targeting multiple genes in most of the enriched pathways, 7 of which were up-regulated (let-7c-5p, miR-21-5p, miR-34a-5p, miR-125b-5p, miR-155-5p, miR-193b-3p, miR-218-5p) and 8 down-regulated (let-7a-5p, let-7e-5p, let-7g-5p, miR-16-5p, miR-17-5p, miR-20a-5p, mir-26b-5p, and miR-106b-5p), suggesting their critical role in disease development. Key target genes of identified EV-miRs are listed in Tables S6 and S7. They mainly code for cytokine/chemokines, signaling molecules, transcription factors, cell-cycle and apoptosis regulators, and stress factors, such as SOCS1, TNF, VEGFA, IL-6, EGFR, IGF1R, TGFBR2, CCL5, several members of the MAPK and JAK/STAT families, Akt1, mTOR, MYC, NFKB1, SMAD4, DNMT1, CCND1, BCL2, SIRT1, and HMGB1.

Differentially expressed EV-miRs in SF and PL specimens were validated by qRT-PCRs. To this end, we selected five EV-miRs (two up- and three down-regulated) among those listed in Tables 6 and 7 and compared CT values obtained with the Array Cards and qRT-PCR between the two sample groups in 3 representative patients. As depicted in Figure S5, a 100% concordance was detected between the two analyses with respect to the direction of the expression changes, confirming let-7c-5p and miR-21-5p overexpression (lower CT values) and let-7a-5p, let-7g-5p, miR-16-5p downregulation (higher CT values) in SF compared to paired PL samples.

These results characterize the biological processes regulated by EV-miRs within the joints of OJIA patients at an early stage of the disease and identify the EV-miRs that may contribute the most to disease pathogenesis and, thus, represent new potential biomarkers of its development.

## EV-miR expression profiles differentiate new-onset OJIA patients from control children

To identify EV-miRs that could represent early putative diagnostic biomarkers in OJIA, we compared the miRNA expression profile of EVs isolated from SF and PL of new-onset OJIA patients with that of PL samples collected from 8 age- and gender-matched control children (CTR-PL) (Table S1). An average number of 163 out of the 381 EV-miRs analyzed by the Array cards was detectable in the CTR-PL samples.

As shown in Figure 4a, PCA demonstrated a clear separation between OJIA-SF and CTR-PL on the basis of their EV-miRs expression levels, that was confirmed by unsupervised hierarchical clustering analysis (Fig.4b). Differential expression analysis identified a total of 54 EV-miRs, of which 25 were increased and 29 decreased in OJIA-SF respect to CTR-PL (Table S4). Fifteen up- and all down-regulated EV-miRs were the same identified upon comparison of OJIA-SF with paired OJIA-PL samples (Table S4), confirming the existence of a SF-specific EV-miR expression signature.

As reported in Figures 4c and 4d, a marked separation between patients and control children was also observed when EV-miR levels were compared between PL samples (with the exception of CTR-PL 6 and OJIA-PL 9), with 106 EV-miRs significantly upregulated in OJIA-PL *vs* CTR-PL and 4 EV-miRs significantly downregulated (Table S8), suggesting specific disease-related changes in the expression levels of circulating EV-miRs. About 50% overexpressed EV-miRs exhibited a FC ≥4 and an adjusted p value ≤0.01, several of which have been reported to play an important role in inflammatory processes and joint metabolism^27, 82^. The potential confounding effect of age and sex (male vs female) on the observed differences in EV-miR levels between OJIA and CTR subjects was statistically excluded by the analysis of their association with subject group (OJIA vs CTR) using a Student’s t test (for age) and a Fisher’s exact test (for sex) (p>0.05).We then evaluated whether some of the EV-miRs deregulated in OJIA-SF *vs* CTR-PL would overlap with those observed in OJIA-PL vs CTR-PL and allow to derive a common EV-miRs signature with diagnostic potential. Interestingly, as depicted by the Venn diagrams in Figure 4e, a subset of 39 EV-miRs was differentially modulated in both SF and PL samples from OJIA patients respect to control samples, 16 of which (e.g. let-7c-5p, miR-21-5p, miR-24-3p, miR-210-3p, miR-146b-5p, miR-186-5p, miR-345-5p, and miR-590-5p) displayed consensual upregulation. ROC curves generated for each of the 16 upregulated EV-miRs (Fig.4f) showed a clear visual discriminating power of their expression levels between OJIA and CTRL groups. The AUC values computed from the ROC curves ranged from 0.81 to 1.0 between OJIA-SF and CTR-PL and from 0.83 to 1.0 between OJIA-PL and CTR-PL (Table S9), indicating a high potential diagnostic value of all modulated EV-miRs. qRT-PCR analysis confirmed higher expression levels of four randomly selected EV-miRs, such as let-7c-5p, miR-146b-5p, miR-210-3p, and miR-21-3p, in PL and SF samples isolated from three representative OJIA patients compared to PL samples from three control subjects, showing a 100% concordance with data obtained with the Array Cards (Fig.S6).

Taken together, these data demonstrate that the expression levels of several EV-miRs can differentiate new-onset OJIA patients from control children, representing a disease-specific molecular “fingerprint” with diagnostic potential.

# DISCUSSION

In the last years, a large number of studies has demonstrated the value of miRNA profiling in EVs released into body fluids as an efficient approach to elucidate the pathogenic mechanisms of adult rheumatic diseases and discover novel biomarkers for clinical use ^15, 33^. However, no data were reported on EV-miR expression profiles in OJIA, and their potential as biomarkers has not been explored. In this work, we provide an optimized procedure for EV-miR profiling in small volumes of SF and the first comparative evaluation of EV-miR expression patterns in SF and PL samples from OJIA patients at disease onset. Our results define EV-miR signatures potentially implicated in disease pathogenesis and able to discriminate new-onset OJIA patients from healthy children, yielding a framework for the identification of early EV-miR-based biomarkers of disease development and diagnosis.

EV-miR profiling faces various technical challenges related to sample collection and handling, EV isolation, and EV-miR purification and detection^65, 83, 84^. Several pre-analytical variables as well as biofluid-specific contaminants can affect both EV recovery and EV-RNA analysis^59, 60^. This implies that sample processing conditions need to be tailored to each specific type of fluid and intended downstream applications. Most of the studies reported in the literature characterize EV-miRs in plasma or serum samples^83, 85, 86^, and, to our knowledge, no standardized protocol is currently available for the analysis of EV-miR expression profile in SF specimens. As a complex biofluid, SF suffers from limitations for EV purification using traditional differential ultracentrifugation-based methods (e.g. high contamination with non-vesicular materials, vesicles rupture or artifactual aggregation, low recovery of small EVs and poor EV-miR purification from little sample amounts), thus requiring different approaches ^87, 88^. EVs were isolated from a small volume of SF samples from new-onset OJIA patients using a membrane affinity spin column-based procedure previously described as a reliable method to collect intact EVs from plasma samples and to extract their RNA content with high yield and purity while excluding non-vesicular RNA ^41, 89^. We demonstrated the efficient enrichment of a relatively monodisperse population of particles with morphology and size-range consistent with both exosomes and microvesicles and expressing high levels of the typical EV markers, CD9 and CD63. The lack of CD81 expression observed in the EV preparations is in line with earlier publications showing that the membrane affinity technique primarily isolates a CD81- EV subpopulation with larger sizes respect to other isolation methods from different types of biological samples ^11,90-93^.

The apparent discrepancies in particle size distribution observed among TEM, DLS, and NTA were expected given the different technical features of these methods. TEM allows to distinguish and measure each single particle within a mixed population, whereas DLS cannot accurately resolve heterogeneous mixtures because it measures instantaneously the intensity of the light scattered from a bulk of particles present in an aqueous sample, which is proportional to the sixth power of their diameter, being thus biased towards larger particles and shifting size distribution towards higher values^43, 94^. In addition, because DLS assumes any aggregation of several vesicles as one single particle, it is also probable that some aggregates present in the samples, which was excluded in size quantification by TEM but could not be eliminated by DLS measurements, have contributed to the apparent EV size overestimation by DLS respect to TEM^43, 94^. The differences observed in small EV sizing by NTA respect to TEM can be explained by the fact that particles <70 nm may be underestimated by NTA, falling below the minimum detectable particle size for the technique (46–70 nm depending on the refractive index), and that NTA performance is still influenced by the intensity of the light scattering of large particles although in a lower rate respect to DLS^94^.

Moreover, due to the different procedure of sample preparation for DLS/NTA and TEM, particles are analyzed in a hydrated and dehydrated state, respectively, which may also contribute to size over- and under-estimation, as reported previously^95, 96^. However, because the membrane affinity column used for EV isolation cannot completely rid complex biofluid samples of lipoproteins, which might co-isolate in EV fractions^90-92^, a potential contribution of lipoprotein particles to the overall size differences observed between DLS/NTA and TEM cannot be ruled out.

Divergent results have been reported on the impact of various anticoagulants on EV recovery from PL samples, with the use of heparin-based protease inhibitors and calcium-chelating agents recommended in some studies and discouraged in others ^59, 60, 97^. However, the effects of anticoagulant on EV isolation from SF samples have not been assessed. In addition, although the interference of some anticoagulants with nucleic acid analysis by RT-qPCR has been demonstrated ^49, 61^, to our knowledge no study has analyzed to which extent they affect the profile of miRNAs encapsulated in EVs. We found substantially comparable EV yield and morphological characteristics in SF samples collected into EDTA and sodium-heparin tubes. In contrast, miRNA detection in EVs significantly differed depending on the type of anticoagulant used. High quality small-RNA profile and EV-miR amplification were obtained from EDTA samples, whereas a non-conventional small-RNA profile and lack of amplification were observed in those collected into sodium-heparin tubes. RNA treatment with heparinase I improved small-RNA quality and yielded successful amplification, demonstrating that heparin can interfere with the analysis of miRNAs contained in EVs similarly to what reported for free circulating miRNAs ^49, 61^ and that the de-heparinization procedure provides a mean for EV-miR assessment in SF samples when the use of heparin is unavoidable. However, despite de-heparination, lower efficiency in EV-miR detection was obtained upon sample collection in heparin respect to EDTA both in terms of absolute number and expression levels. SF is characterized by a complex biological matrix which can negatively affect EV isolation ^98^. Previous studies by Boere et al. have shown that SF treatment with HYase may facilitate EV isolation from SF by breaking down hyaluronan, chondroitin sulphate, and keratin sulfate, associated with a decrease in protein-matrix interactions ^63^. Consistent with these findings, we demonstrated that HYase pretreatment improved EV recovery and increased EV-miR detection in SF samples collected in EDTA tubes. Taken together, these data strongly suggest that the use of EDTA in combination with HYase offers the best performance for the characterization of miRNA profiles in EVs isolated from human inflammatory SF.

Using this procedure, we detected in OJIA-SF samples several EV-miRs previously associated with the pathogenesis of rheumatoid arthritis (RA)^27, 28, 68^, osteoarthritis (OA)^99, 100^, systemic and polyarticular JIA^30, 31^ and regarded as potential biomarkers and/or new targets of treatment in these diseases. A few identified EV-miRs, such as miR146b-5p, miR-16-5p, miR-150-5p, miR-223-3p, miR-155-5p, and miR-146a-5p, have been previously found in the joints of arthritic patients either as free molecules released into the SF^27, 28, 39^ or in cells of the synovial tissue^101-103^, and our findings extend these results demonstrating their presence also in SF-derived EVs. These data highlight the suitability of our experimental protocol for EV-miR profiling in small volumes of SF, providing an efficient mean for the identification of new SF-derived biomarkers in OJIA.

Given the role of miRNAs in immune and inflammatory responses ^23, 24, 104^, it is conceivable that their deregulation within the joints may be critical for disease pathogenesis. In this respect, we demonstrated that the EV-miR profile in SF from OJIA patients differed significantly from that of paired PL samples upon disease initial presentation, with the expression levels of 24 and 79 miRNAs up- and down-regulated, respectively. Expression changes were confirmed by qRT-PCR performed on selected EV-miRs. The observed EV-miR differences may reflect the enrichment of distinct EVs subpopulations endowed with specific biologic functions or of different nature^16, 67, 105^ in the joints respect to the peripheral circulation, related to the diverse cellular composition of the two biologic fluids. This conclusion is supported by our previous findings highlighting the different miRNA profiles of mononuclear cells isolated from the SF and PB of OJIA patients ^106^. Distinct patterns of EV-miRs in SF *vs* PL samples have been previously reported by Murata et al. ^28^ in adult arthritides (RA and OA). Interestingly, a few miRNAs, such as miR-16-5p and miR-223-3p, were similarly downregulated in OJIA-SF (this study), RA-SF, and OA-SF^28^ respect to paired PL samples, whereas others (miR-132-3p, miR-155-5p, and miR-146a-5p) were differentially modulated. These findings provide evidence of both common and specific changes in the miRNA expression pattern of inflamed joints in juvenile and adult forms of arthritis, which may reflect different underlying pathophysiologic processes. Although OJIA shares some similarities with RA ^3, 107^, it has fundamental differences in clinical features and prognosis, representing a completely separate entity unique to the pediatric population whose molecular bases are not yet well understood ^108^. Alternatively, the observed differences between our and Murata’s findings could be attributable to the diverse experimental approach used and the type of miRNAs analyzed. Murata et al. evaluated the expression levels of selected miRNAs freely circulating in the fluids, whereas we analyzed the whole expression profile of miRNAs encapsulated in EVs. In this respect, free-circulating miRNAs were reported to be more prone to variations in expression levels than EV-associated ones probably because of lower stability and, thus, there might be only a limited correlation in their profiles ^21, 35^.

Results of pathway analysis carried out on the genes targeted by EV-miRs differentially expressed in SF vs PL samples revealed their involvement in biologic pathways critical for the normal metabolism of joint tissues but whose deregulation may contribute to arthritis development and progression, such as inflammation and immune responses, inflammatory signaling pathways, cartilage/bone homeostasis, and cell death and damage ^71, 73, 75, 78^. A subset of 15 EV-miRs (7 up- and 8 down-regulated) seemed particularly relevant for OJIA pathogenesis because they targeted multiple genes critically involved in most of the enriched pathways, many of which were shared by different miRNAs suggesting coordinated regulatory activities. According to the literature, 5 of the 7 upregulated EV-miRs (miR-155-5p, miR-125b-5p, miR-21-5p, miR-34a-5p, and miR-218-5p) are endowed with pro-inflammatory and/or pro-apoptotic functions ^109-111^ and were reported to play a crucial role in various chronic inflammatory and autoimmune disorders, including rheumatic diseases ^112, 113^. They were found overexpressed in synovial tissues (ST), SF, and cartilage of OA and RA patients and considered as biomarkers in these diseases ^114-116^, contributing to fibroblast-like synoviocyte (FLS) and chondrocyte proliferation, apoptosis, and destructive activity ^99, 101, 115, 117^, synovial inflammation ^114, 116, 118^, and cartilage degradation^115, 119^. Conversely, the 8 down-regulated EV-miRs (miR-17-5p, miR-20a-5p, miR-16-5p, let-7a-5p, let-7g-5p, miR-26b-5p, Let-7e-5p and miR-106b-5p) exhibit anti-inflammatory and anti-erosive activities ^120-123^ and were detected at lower levels in SF/ST and cartilage tissues from RA and OA patients respect to healthy controls ^82, 124, 125^. miR-155, miR-21, miR-125b, and miR-34a ^114, 126-128^, as well as miR-16-5p miR-26b-5p, and let7g-5p ^82, 120, 129^, were also implicated in the disruption of Th17/Treg balance, which is critical for arthritis pathogenesis ^130^. Interestingly, inhibition of up-regulated miRNAs by specific antagomirs ^115, 117, 119^ in pre-clinical animal models or treatment with mimics of downregulated miRNAs ^82, 124, 131^ were shown to attenuate arthritis manifestation and progression. These data define the potential mechanisms by which SF-EVs may contribute to OJIA pathophysiology and identify a panel of EV-miRs that are likely to play a critical role in the promotion of synovitis and joint damage on the one hand and in the inhibition of the processes involved in the resolution of inflammation on the other, highlighting their potential as novel biomarkers of OJIA development and targets of therapy. However, because pathway analysis is biased towards well-studied miRNAs, it is possible that other EV-miRs with pathophysiologic relevance were not identified given to their poor characterization in the literature.

We have previously demonstrated that the hypoxic synovial microenvironment is critical for OJIA pathogenesis, contributing to the amplification of inflammatory responses and dysregulation of angiogenesis ^3, 132^. Hypoxia is known to affect the expression and function of a panel of miRNAs, which in turn can regulate cell adaptive response to reduced oxygenation ^133, 134^ by targeting the hypoxia-inducible transcription factors (HIF)-1α/2α, two major players in inflammation, angiogenesis, cartilage and bone remodeling ^3, 135^. Interestingly, we found that 12 of the 15 differentially expressed EV-miRs (let-7c, miR-21-5p, miR-34a-5p, miR-155-5p, miR-193b-3p, and miR-218-5p among up-regulated and miR-17-5p, miR-20a-5p, miR-16-5p, let-7a-5p, miR-26b-5p, and let-7e-5p among downregulated) targeted genes involved in cell response to hypoxia, some of which have been previously identified as hypoxiamirs in a tissue- or cell type-specific manner. In particular, the pro-inflammatory miR-155-5p was shown to be upregulated by hypoxia in epithelial and endothelial cells via direct HIF-1α binding to a hypoxia response elements located in its promoter and to control HIF-1α mRNA expression and transcriptional activity ^136, 137^. On the other hand, the anti-inflammatory miR-16-5p, miR-17-5p, miR-20a-5p, and miR-26b-5p are negatively regulated by hypoxia via a HIF-dependent mechanism ^138, 139^, and their decreased expression was reported to upregulate HIF-1α,-2α ^140^ with important effects on cell apoptosis^141^, osteoclast differentiation^142^, and macrophage proangiogenic functions^143, 144^. These data suggest that hypoxia pathogenic effects are controlled at least in part through EV-miR deregulation in the joints.

Pathway analysis also revealed the significant enrichment in OJIA-SF of miRNA target genes implicated in sex hormone and insulin metabolism/signaling processes. Sex hormones have profound influence on immune system development/function and inflammatory responses and were reported to contribute to the pathogenesis of autoimmune diseases via miRNA dysregulation ^104^. Their altered balance in the synovium has been implicated in the initiation and amplification of synovial inflammation, hyperplasia, and damage, contributing to the onset and progression of various rheumatic disorders ^79, 80, 145^. Insulin resistance was also shown to correlate significantly with markers of inflammation and to be higher in patients with RA than in the general population ^146^, and insulin-like growth factor 1 (IGF-1) abnormalities were observed in children with chronic inflammatory conditions, including JIA ^81, 147^. miR-155-5p, miR-17-5p, miR-21-5p, and miR-16-5p, among others, have been shown to mediate the link between inflammation and insulin sensitivity in children with chronic inflammatory diseases ^81, 120^. The association between hormone profiles and altered EV-miR expression levels in the joint of OJIA patients is noteworthy and deserves further investigation.

Recent studies have reported deregulated expression of free-circulating miRNAs in systemic and polyarticular JIA subtypes and proposed them as diagnostic biomarkers for these diseases ^30, 31, 148^. Our study identifies for the first time a panel of EV-miRs whose expression levels in PL samples could effectively differentiate OJIA patients at disease onset from age-matched control children. Specifically, 110 EV-miRs significantly differed between the two groups of children, among which 106 were upregulated and 4 downregulated in patients relative to controls, and expression changes of selected EV-miRs were confirmed by qRT-PCR analysis. Four of upregulated miRNAs, such as miR-146a-5p, miR-26a-5p, miR-145-5p, and miR-181a-5p, have been found overexpressed also in the PL of patients affected by other JIA subtypes and shown to correlate with disease clinical parameters ^31, 148, 149^, probably representing common biomarkers in different forms of JIA. In contrast, no previous studies in JIA have reported alterations in the levels of the other EV-miRs that we found differentially expressed in diseased *vs* control children, neither as free-circulating nor as EV-associated molecules, raising the possibility that they may be specific for the OJIA subtype. Conversely, altered circulating levels of a set of these miRNAs (e.g. mir-21-5p, let-7a-5p, miR-24-3p, miR-26a-5p, miR-335-5p, and mir-125a-5p), have been observed in RA and/or OA patients ^27, 150, 151^, indicating their potential relevance as diagnostic biomarkers in both juvenile and adult forms of arthritis.

Importantly, we defined a large group of EV-miRs significantly modulated (FC ≥4; adjusted p value ≤0.01) in the PL of OJIA children which have not been previously described in the circulation of patients affected by any other arthritic condition. Among them, miR-345-5p, miR-590-5p, miR-139-3p, miR-186-5p, and miR-411-5p are particularly attractive as new putative biomarkers in OJIA given to their roles in inflammatory responses ^152, 153^ and chondrocyte/FLS activities ^152, 154-156^. Particularly intriguing is also the finding that a subset of 16 EV-miRs, which comprises both miRNAs previously reported as markers in rheumatic diseases (e.g.miR-21-5p, miR-24-3p, miR-132-3p, miR-146a-5p, and let-7c-5p) and newly identified molecules (e.g.miR-186-5p, miR-345-5p, and miR-590-5p), was consensually overexpressed and displayed a high discriminating profile in both PL and SF samples from new-onset OJIA patients respect to CTR-PL specimens, highlighting the possibility that it may represent a specific disease-associated EV-miR signature measurable at both the local and systemic level with important diagnostic potential in OJIA.

In conclusion, this is the first study that comprehensively characterizes the miRNome of EVs isolated from OJIA patients, representing a significant step forward in the understanding of the molecular basis of OJIA and of the role and mechanism of action of EV-miRs in joint inflammation and destruction. A limitation of this work is the lack of comparison of EV-miR expression levels between SF samples from OJIA patients and SF from control subjects. Unfortunately, this issue cannot be addressed due to ethical reasons. Another drawback is represented by the small sample size analyzed. A prospective study is currently ongoing to validate these results in a larger cohort of OJIA patients and to determine the correlation of the identified EV-miR-based signatures with patient clinical parameters and their specificity for OJIA through the comparison with samples isolated from patients with other types of JIA. Despite these limitations, our findings demonstrate for the first time that EV-miR levels could represent new molecular indicators of OJIA development and identify a panel of EV-miRs able to discriminate OJIA patients from healthy children at disease onset, with important clinical implications. Assessment of EV-miR biomarker may, in fact, contribute to earlier disease diagnosis in suspect patients and allow to initiate appropriate therapy at disease presentation, thus increasing the probability to limit the occurrence of joint damage and preserve its functionality and minimizing patient exposure to the potential side-effects of ineffective medications, with clear benefit to patients. In addition. these data provide the bases for investigating the potential of EV-miRs as targets of novel tailored therapeutic strategies in OJIA, paving the way to personalized medicine.

# REFERENCES

# Martini A, Lovell DJ. Juvenile idiopathic arthritis: state of the art and future perspectives. Ann Rheum Dis 2010;69(7):1260-1263.

# Macaubas C, Nguyen K, Milojevic D, Park JL, Mellins ED. Oligoarticular and polyarticular JIA: epidemiology and pathogenesis. Nat Rev Rheumatol 2009;5(11):616-626.

# Bosco M.C., Varesio L. Monocytic Cell Gene Regulation by the Hypoxic Synovial Environment in Juvenile Idiopathic Arthritis: Implications for Disease Pathogenesis. Journal of Clinical Rheumatology and Musculoskeletal Medicine 2010;1(1):47-55.

# Ravelli A, Davi S, Bracciolini G et al. Intra-articular corticosteroids versus intra-articular corticosteroids plus methotrexate in oligoarticular juvenile idiopathic arthritis: a multicentre, prospective, randomised, open-label trial. Lancet 2017;389(10072):909-916.

# Schiappapietra B, Bava C, Rosina S et al. A prediction rule for polyarticular extension in oligoarticular-onset juvenile idiopathic arthritis. Clin Exp Rheumatol 2021;39(4):913-919.

# Consolaro A, Varnier GC, Martini A, Ravelli A. Advances in biomarkers for paediatric rheumatic diseases. Nat Rev Rheumatol 2015;11(5):265-275.

# Duurland CL, Wedderburn LR. Current developments in the use of biomarkers for juvenile idiopathic arthritis. Curr Rheumatol Rep 2014;16(3):406.

# Hunter PJ, Nistala K, Jina N et al. Biologic predictors of extension of oligoarticular juvenile idiopathic arthritis as determined from synovial fluid cellular composition and gene expression. Arthritis Rheum 2010;62(3):896-907.

# Gibson DS, Finnegan S, Jordan G et al. Stratification and monitoring of juvenile idiopathic arthritis patients by synovial proteome analysis. J Proteome Res 2009;8(12):5601-5609.

# Tu ZQ, Xue HY, Chen W, Cao LF, Zhang WQ. Identification of potential peripheral blood diagnostic biomarkers for patients with juvenile idiopathic arthritis by bioinformatics analysis. Rheumatol Int 2017;37(3):423-434.

# Lipps C, Northe P, Figueiredo R et al. Non-Invasive Approach for Evaluation of Pulmonary Hypertension Using Extracellular Vesicle-Associated Small Non-Coding RNA. Biomolecules 2019;9(11).

# De TJ, Herschlik L, Waldner C, Mongini C. Emerging roles of exosomes in normal and pathological conditions: new insights for diagnosis and therapeutic applications. Front Immunol 2015;6:203.

# Shah R, Patel T, Freedman JE. Circulating Extracellular Vesicles in Human Disease. N Engl J Med 2018;379(10):958-966.

# Wu WC, Song SJ, Zhang Y, Li X. Role of Extracellular Vesicles in Autoimmune Pathogenesis. Front Immunol 2020;11:579043.

# Tavasolian F, Moghaddam AS, Rohani F et al. Exosomes: Effectual players in rheumatoid arthritis. Autoimmun Rev 2020;19(6):102511.

# Foers AD, Cheng L, Hill AF, Wicks IP, Pang KC. Review: Extracellular Vesicles in Joint Inflammation. Arthritis Rheumatol 2017;69(7):1350-1362.

# Meldolesi J. Extracellular vesicles, news about their role in immune cells: physiology, pathology and diseases. Clin Exp Immunol 2019;196(3):318-327.

# Robbins PD, Dorronsoro A, Booker CN. Regulation of chronic inflammatory and immune processes by extracellular vesicles. J Clin Invest 2016;126(4):1173-1180.

# Xu R, Greening DW, Zhu HJ, Takahashi N, Simpson RJ. Extracellular vesicle isolation and characterization: toward clinical application. J Clin Invest 2016;126(4):1152-1162.

# van NG, D'Angelo G, Raposo G. Shedding light on the cell biology of extracellular vesicles. Nat Rev Mol Cell Biol 2018;19(4):213-228.

# Gallo A, Tandon M, Alevizos I, Illei GG. The majority of microRNAs detectable in serum and saliva is concentrated in exosomes. PLoS One 2012;7(3):e30679.

# Valadi H, Ekstrom K, Bossios A, Sjostrand M, Lee JJ, Lotvall JO. Exosome-mediated transfer of mRNAs and microRNAs is a novel mechanism of genetic exchange between cells. Nat Cell Biol 2007;9(6):654-659.

# Baltimore D, Boldin MP, O'Connell RM, Rao DS, Taganov KD. MicroRNAs: new regulators of immune cell development and function. Nat Immunol 2008;9(8):839-845.

# Xiao C, Rajewsky K. MicroRNA control in the immune system: basic principles. Cell 2009;136(1):26-36.

# Quinn JF, Patel T, Wong D et al. Extracellular RNAs: development as biomarkers of human disease. J Extracell Vesicles 2015;4:27495.

# Zeng L, Cui J, Wu H, Lu Q. The emerging role of circulating microRNAs as biomarkers in autoimmune diseases. Autoimmunity 2014;47(7):419-429.

# Churov AV, Oleinik EK, Knip M. MicroRNAs in rheumatoid arthritis: altered expression and diagnostic potential. Autoimmun Rev 2015;14(11):1029-1037.

# Murata K, Yoshitomi H, Tanida S et al. Plasma and synovial fluid microRNAs as potential biomarkers of rheumatoid arthritis and osteoarthritis. Arthritis Res Ther 2010;12(3):R86.

# Wang H, Peng W, Ouyang X, Li W, Dai Y. Circulating microRNAs as candidate biomarkers in patients with systemic lupus erythematosus. Transl Res 2012;160(3):198-206.

# Kamiya Y, Kawada J, Kawano Y et al. Serum microRNAs as Potential Biomarkers of Juvenile Idiopathic Arthritis. Clin Rheumatol 2015;34(10):1705-1712.

# Ma X, Wu F, Xin L et al. Differential plasma microRNAs expression in juvenile idiopathic arthritis. Mod Rheumatol 2016;26(2):224-232.

# Demir F, Cebi AH, Kalyoncu M. Evaluation of plasma microRNA expressions in patients with juvenile idiopathic arthritis. Clin Rheumatol 2018;37(12):3255-3262.

# Foers AD, Garnham AL, Chatfield S et al. Extracellular Vesicles in Synovial Fluid from Rheumatoid Arthritis Patients Contain miRNAs with Capacity to Modulate Inflammation. Int J Mol Sci 2021;22(9).

# Ge Q, Zhou Y, Lu J, Bai Y, Xie X, Lu Z. miRNA in plasma exosome is stable under different storage conditions. Molecules 2014;19(2):1568-1575.

# Zhang ZY, Li YC, Geng CY, Wang HJ, Chen WM. Potential Relationship between Clinical Significance and Serum Exosomal miRNAs in Patients with Multiple Myeloma. Biomed Res Int 2019;2019:1575468.

# Skog J, Wurdinger T, van RS et al. Glioblastoma microvesicles transport RNA and proteins that promote tumour growth and provide diagnostic biomarkers. Nat Cell Biol 2008;10(12):1470-1476.

# Bhome R, Del VF, Lee GH et al. Exosomal microRNAs (exomiRs): Small molecules with a big role in cancer. Cancer Lett 2018;420:228-235.

# Withrow J, Murphy C, Liu Y, Hunter M, Fulzele S, Hamrick MW. Extracellular vesicles in the pathogenesis of rheumatoid arthritis and osteoarthritis. Arthritis Res Ther 2016;18(1):286.

# Nziza N, Jeziorski E, Delpont M et al. Synovial-Fluid miRNA Signature for Diagnosis of Juvenile Idiopathic Arthritis. Cells 2019;8(12).

# Petty RE, Southwood TR, Manners P et al. International League of Associations for Rheumatology classification of juvenile idiopathic arthritis: second revision, Edmonton, 2001. J Rheumatol 2004;31(2):390-392.

# Enderle D, Spiel A, Coticchia CM et al. Characterization of RNA from Exosomes and Other Extracellular Vesicles Isolated by a Novel Spin Column-Based Method. PLoS One 2015;10(8):e0136133.

# Marimpietri D, Petretto A, Raffaghello L et al. Proteome profiling of neuroblastoma-derived exosomes reveal the expression of proteins potentially involved in tumor progression. PLoS One 2013;8(9):e75054.

# Danaei M, Dehghankhold M, Ataei S et al. Impact of Particle Size and Polydispersity Index on the Clinical Applications of Lipidic Nanocarrier Systems. Pharmaceutics 2018;10(2).

# Santamaria S, Gagliani MC, Bellese G et al. Imaging of Endocytic Trafficking and Extracellular Vesicles Released Under Neratinib Treatment in ERBB2(+) Breast Cancer Cells. J Histochem Cytochem 2021;69(7):461-473.

# Bachurski D, Schuldner M, Nguyen PH et al. Extracellular vesicle measurements with nanoparticle tracking analysis - An accuracy and repeatability comparison between NanoSight NS300 and ZetaView. J Extracell Vesicles 2019;8(1):1596016.

# Mitra A, Yoshida-Court, Solley TN et al. Extracellular vesicles derived from ascitic fluid enhance growth and migration of ovarian cancer cells. Sci Rep 2021;11(1):9149.

# Cilibrasi C, Simon T, Vintu M et al. Definition of an Inflammatory Biomarker Signature in Plasma-Derived Extracellular Vesicles of Glioblastoma Patients. Biomedicines 2022;10(1).

# Hsu CY, Hsieh TH, Lin HY et al. Characterization and Proteomic Analysis of Endometrial Stromal Cell-Derived Small Extracellular Vesicles. J Clin Endocrinol Metab 2021;106(5):1516-1529.

# Kondratov K, Kurapeev D, Popov M et al. Heparinase treatment of heparin-contaminated plasma from coronary artery bypass grafting patients enables reliable quantification of microRNAs. Biomol Detect Quantif 2016;8:9-14.

# Resaz R, Cangelosi D, Segalerba D et al. Exosomal MicroRNAs as Potential Biomarkers of Hepatic Injury and Kidney Disease in Glycogen Storage Disease Type Ia Patients. Int J Mol Sci 2021;23(1).

# Zanardi N, Morini M, Tangaro MA et al. PIPE-T: a new Galaxy tool for the analysis of RT-qPCR expression data. Sci Rep 2019;9(1):17550.

# Liew AW, Law NF, Yan H. Missing value imputation for gene expression data: computational techniques to recover missing data from available information. Brief Bioinform 2011;12(5):498-513.

# Mestdagh P, Van VP, De WA et al. A novel and universal method for microRNA RT-qPCR data normalization. Genome Biol 2009;10(6):R64.

# Metsalu T, Vilo J. ClustVis: a web tool for visualizing clustering of multivariate data using Principal Component Analysis and heatmap. Nucleic Acids Res 2015;43(W1):W566-W570.

# Heberle H, Meirelles GV, da Silva FR, Telles GP, Minghim R. InteractiVenn: a web-based tool for the analysis of sets through Venn diagrams. BMC Bioinformatics 2015;16(1):169.

# Sticht C, De La Torre C, Parveen A, Gretz N. miRWalk: An online resource for prediction of microRNA binding sites. PLoS One 2018;13(10):e0206239.

# Chang L, Zhou G, Soufan O, Xia J. miRNet 2.0: network-based visual analytics for miRNA functional analysis and systems biology. Nucleic Acids Res 2020;48(W1):W244-W251.

# Goksuluk D, Korkmaz, S. et al. easyROC: An Interactive Web-tool for ROC Curve Analysis Using R Language Environment PDF download. The R Journal 2016;8(2):213-230.

# Baek R, Sondergaard EK, Varming K, Jorgensen MM. The impact of various preanalytical treatments on the phenotype of small extracellular vesicles in blood analyzed by protein microarray. J Immunol Methods 2016;438:11-20.

# Jayachandran M, Miller VM, Heit JA, Owen WG. Methodology for isolation, identification and characterization of microvesicles in peripheral blood. J Immunol Methods 2012;375(1-2):207-214.

# Moldovan L, Batte KE, Trgovcich J, Wisler J, Marsh CB, Piper M. Methodological challenges in utilizing miRNAs as circulating biomarkers. J Cell Mol Med 2014;18(3):371-390.

# Fam H, Bryant JT, Kontopoulou M. Rheological properties of synovial fluids. Biorheology 2007;44(2):59-74.

# Boere J, van de Lest CH, Libregts SF et al. Synovial fluid pretreatment with hyaluronidase facilitates isolation of CD44+ extracellular vesicles. J Extracell Vesicles 2016;5:31751.

# GyÃ¶rgy B, MÃ³dos K, PÃ¡llinger E et al. Detection and isolation of cell-derived microparticles are compromised by protein complexes resulting from shared biophysical parameters. Blood 2011;117(4):e39-e48.

# Willms E, CabaÃ±as C, MÃ¤ger I, Wood MJA, Vader P. Extracellular Vesicle Heterogeneity: Subpopulations, Isolation Techniques, and Diverse Functions in Cancer Progression. Front Immunol 2018;9:738.

# Kowal J, Arras G, Colombo M et al. Proteomic comparison defines novel markers to characterize heterogeneous populations of extracellular vesicle subtypes. Proc Natl Acad Sci U S A 2016;113(8):E968-E977.

# Zabeo D, Cvjetkovic A, LÃ¤sser C, Schorb M, LÃ¶tvall J, HÃ¶Ã¶g JL. Exosomes purified from a single cell type have diverse morphology. J Extracell Vesicles 2017;6(1):1329476.

# Guggino G, Orlando V, Saieva L et al. Downregulation of miRNA17-92 cluster marks VÎ³9VÎ´2 T cells from patients with rheumatoid arthritis. Arthritis Res Ther 2018;20(1):236.

# Xie Y, Zinkle A, Chen L, Mohammadi M. Fibroblast growth factor signalling in osteoarthritis and cartilage repair. Nat Rev Rheumatol 2020;16(10):547-564.

# Vomero M, Barbati C, Colasanti T et al. Autophagy and Rheumatoid Arthritis: Current Knowledges and Future Perspectives. Front Immunol 2018;9:1577.

# Finnson KW, Chi Y, Bou-Gharios G, Leask A, Philip A. TGF-b signaling in cartilage homeostasis and osteoarthritis. Front Biosci (Schol Ed) 2012;4:251-268.

# Muz B, Khan MN, Kiriakidis S, Paleolog EM. Hypoxia. The role of hypoxia and HIF-dependent signalling events in rheumatoid arthritis. Arthritis Res Ther 2009;11(1):201.

# Sun K, Luo J, Guo J, Yao X, Jing X, Guo F. The PI3K/AKT/mTOR signaling pathway in osteoarthritis: a narrative review. Osteoarthritis Cartilage 2020;28(4):400-409.

# Gao X, Sun Y, Li X. Identification of key gene modules and transcription factors for human osteoarthritis by weighted gene co-expression network analysis. Exp Ther Med 2019;18(4):2479-2490.

# Zhou Y, Wang T, Hamilton JL, Chen D. Wnt/Î²-catenin Signaling in Osteoarthritis and in Other Forms of Arthritis. Curr Rheumatol Rep 2017;19(9):53.

# Malemud CJ. Negative Regulators of JAK/STAT Signaling in Rheumatoid Arthritis and Osteoarthritis. Int J Mol Sci 2017;18(3).

# da Fonseca LJS, Nunes-Souza V, Goulart MOF, Rabelo LA. Oxidative Stress in Rheumatoid Arthritis: What the Future Might Hold regarding Novel Biomarkers and Add-On Therapies. Oxid Med Cell Longev 2019;2019:7536805.

# Baier A, Meineckel I, Gay S, Pap T. Apoptosis in rheumatoid arthritis. Curr Opin Rheumatol 2003;15(3):274-279.

# AlpÃ­zar-RodrÃ­guez D, Pluchino N, Canny G, Gabay C, Finckh A. The role of female hormonal factors in the development of rheumatoid arthritis. Rheumatology (Oxford) 2017;56(8):1254-1263.

# Borba VV, Zandman-Goddard G, Shoenfeld Y. Prolactin and autoimmunity: The hormone as an inflammatory cytokine. Best Pract Res Clin Endocrinol Metab 2019;33(6):101324.

# Cirillo F, Lazzeroni P, Catellani C, Sartori C, Amarri S, Street ME. MicroRNAs link chronic inflammation in childhood to growth impairment and insulin-resistance. Cytokine Growth Factor Rev 2018;39:1-18.

# Yang P, Zhang M, Wang X et al. MicroRNA let-7g-5p alleviates murine collagen-induced arthritis by inhibiting Th17 cell differentiation. Biochem Pharmacol 2020;174:113822.

# Mateescu B, Kowal EJ, van Balkom BW et al. Obstacles and opportunities in the functional analysis of extracellular vesicle. J Extracell Vesicles 2017;6(1):1286095.

# Van DJ, Mestdagh P, Sormunen R et al. The impact of disparate isolation methods for extracellular vesicles on downstream RNA profiling. J Extracell Vesicles 2014;3.

# Andreu Z, Rivas E, Sanguino-Pascual A et al. Comparative analysis of EV isolation procedures for miRNAs detection in serum samples. J Extracell Vesicles 2016;5:31655.

# Muller L, Hong CS, Stolz DB, Watkins SC, Whiteside TL. Isolation of biologically-active exosomes from human plasma. J Immunol Methods 2014;411:55-65.

# Linares R, Tan S, Gounou C, Arraud N, Brisson AR. High-speed centrifugation induces aggregation of extracellular vesicles. J Extracell Vesicles 2015;4:29509.

# Foers AD, Chatfield S, Dagley LF et al. Enrichment of extracellular vesicles from human synovial fluid using size exclusion chromatography. J Extracell Vesicles 2018;7(1):1490145.

# El-Khoury V, Pierson S, Kaoma T, Bernardin F, Berchem G. Assessing cellular and circulating miRNA recovery: the impact of the RNA isolation method and the quantity of input material. Sci Rep 2016;6:19529.

# Stranska R, Gysbrechts L, Wouters J et al. Comparison of membrane affinity-based method with size-exclusion chromatography for isolation of exosome-like vesicles from human plasma. J Transl Med 2018;16(1):1.

# Buschmann D, Kirchner B, Hermann S et al. Evaluation of serum extracellular vesicle isolation methods for profiling miRNAs by next-generation sequencing. J Extracell Vesicles 2018;7(1):1481321.

# Brennan K, Martin K, FitzGerald SP et al. A comparison of methods for the isolation and separation of extracellular vesicles from protein and lipid particles in human serum. Sci Rep 2020;10(1):1039.

# Karimi N, Dalirfardouei R, Dias T, LÃ¶tvall J, LÃ¤sser C. Tetraspanins distinguish separate extracellular vesicle subpopulations in human serum and plasma - Contributions of platelet extracellular vesicles in plasma samples. J Extracell Vesicles 2022;11(5):e12213.

# Filipe V, Hawe A, Jiskoot W. Critical evaluation of Nanoparticle Tracking Analysis (NTA) by NanoSight for the measurement of nanoparticles and protein aggregates. Pharm Res 2010;27(5):796-810.

# Chernyshev VS, Rachamadugu R, Tseng YH et al. Size and shape characterization of hydrated and desiccated exosomes. Anal Bioanal Chem 2015;407(12):3285-3301.

# Varga Z, FehÃ©r B, Kitka D et al. Size Measurement of Extracellular Vesicles and Synthetic Liposomes: The Impact of the Hydration Shell and the Protein Corona. Colloids Surf B Biointerfaces 2020;192:111053.

# Gyorgy B, Paloczi K, Kovacs A et al. Improved circulating microparticle analysis in acid-citrate dextrose (ACD) anticoagulant tube. Thromb Res 2014;133(2):285-292.

# Jayadev C, Rout R, Price A, Hulley P, Mahoney D. Hyaluronidase treatment of synovial fluid to improve assay precision for biomarker research using multiplex immunoassay platforms. J Immunol Methods 2012;386(1-2):22-30.

# Ma S, Zhang A, Li X et al. MiR-21-5p regulates extracellular matrix degradation and angiogenesis in TMJOA by targeting Spry1. Arthritis Res Ther 2020 May 1;22 (1):99 doi : 10 1186 /s13075 -020 -2145 -y 2020;22(1):99.

# Xie W, Su W, Xia H, Wang Z, Su C, Su B. Synovial Fluid MicroRNA-210 as a Potential Biomarker for Early Prediction of Osteoarthritis. Biomed Res Int 2019;2019:7165406.

# Stanczyk J, Pedrioli DM, Brentano F et al. Altered expression of MicroRNA in synovial fibroblasts and synovial tissue in rheumatoid arthritis. Arthritis Rheum 2008;58(4):1001-1009.

# Shibuya H, Nakasa T, Adachi N et al. Overexpression of microRNA-223 in rheumatoid arthritis synovium controls osteoclast differentiation. Mod Rheumatol 2013;23(4):674-685.

# Niimoto T, Nakasa T, Ishikawa M et al. MicroRNA-146a expresses in interleukin-17 producing T cells in rheumatoid arthritis patients. BMC Musculoskelet Disord 2010;11:209.

# Dai R, Ahmed SA. MicroRNA, a new paradigm for understanding immunoregulation, inflammation, and autoimmune diseases. Transl Res 2011;157(4):163-179.

# LÃ¤sser C, Jang SC, LÃ¶tvall J. Subpopulations of extracellular vesicles and their therapeutic potential. Mol Aspects Med 2018;60:1-14.

# Rajendiran A, Klemm P, Schippers A et al. miR-23a contributes to T cellular redox metabolism in juvenile idiopathic oligoarthritis. Rheumatology (Oxford) 2021.

# Giancane G, Consolaro A, Lanni S, Davi S, Schiappapietra B, Ravelli A. Juvenile Idiopathic Arthritis: Diagnosis and Treatment. Rheumatol Ther 2016;3(2):187-207.

# Martini A, Ravelli A, Avcin T et al. Toward New Classification Criteria for Juvenile Idiopathic Arthritis: First Steps, Pediatric Rheumatology International Trials Organization International Consensus. J Rheumatol 2019;46(2):190-197.

# Sun YM, Lin KY, Chen YQ. Diverse functions of miR-125 family in different cell contexts. J Hematol Oncol 2013;6:6.

# Alivernini S, Gremese E, McSharry C et al. MicroRNA-155-at the Critical Interface of Innate and Adaptive Immunity in Arthritis. Front Immunol 2017;8:1932.

# Sekar D. Implications of microRNA 21 and its involvement in the treatment of different type of arthritis. Mol Cell Biochem 2021;476(2):941-947.

# Evangelatos G, ", Fragoulis GE, Koulouri V, Lambrou GI, ". MicroRNAs in rheumatoid arthritis: From pathogenesis to clinical impact. Autoimmun Rev 2019;18(11):102391.

# Vicente R, Noel D, Pers YM, Apparailly F, Jorgensen C. Deregulation and therapeutic potential of microRNAs in arthritic diseases. Nat Rev Rheumatol 2016;12(4):211-220.

# Kurowska-Stolarska M, Alivernini S, Ballantine LE et al. MicroRNA-155 as a proinflammatory regulator in clinical and experimental arthritis. Proc Natl Acad Sci U S A 2011;108(27):11193-11198.

# Lu J, Ji ML, Zhang XJ et al. MicroRNA-218-5p as a Potential Target for the Treatment of Human Osteoarthritis. Mol Ther 2017;25(12):2676-2688.

# Zhang B, Wang LS, Zhou YH. Elevated microRNA-125b promotes inflammation in rheumatoid arthritis by activation of NF-ÎºB pathway. Biomed Pharmacother 2017;93:1151-1157.

# Zhang W, Hsu P, Zhong B et al. MiR-34a Enhances Chondrocyte Apoptosis, Senescence and Facilitates Development of Osteoarthritis by Targeting DLL1 and Regulating PI3K/AKT Pathway. Cell Physiol Biochem 2018;48(3):1304-1316.

# Wang Y, Feng T, Duan S et al. miR-155 promotes fibroblast-like synoviocyte proliferation and inflammatory cytokine secretion in rheumatoid arthritis by targeting FOXO3a. Exp Ther Med 2020;19(2):1288-1296.

# Endisha H, Datta P, Sharma A et al. MicroRNA-34a-5p Promotes Joint Destruction During Osteoarthritis. Arthritis Rheumatol 2021;73(3):426-439.

# Yan L, Liang M, Hou X et al. The role of microRNA-16 in the pathogenesis of autoimmune diseases: A comprehensive review. Biomed Pharmacother 2019;112:108583.

# Najm A, Masson FM, Preuss P et al. miR-17-5p reduces inflammation and bone erosions in collagen induced arthritis mice and directly targets the JAK-STAT pathway in rheumatoid arthritis fibroblast-like synoviocytes. Arthritis Rheumatol 2020.

# Zhu W, Yu J, Qiu S et al. MiR-let-7a regulates anti-citrullinated protein antibody-induced macrophage activation and correlates with the development of experimental rheumatoid arthritis. Int Immunopharmacol 2017;51:40-46.

# Feng L, Feng C, Wang CX et al. Circulating microRNA letâ€‘7e is decreased in knee osteoarthritis, accompanied by elevated apoptosis and reduced autophagy. Int J Mol Med 2020;45(5):1464-1476.

# Hu J, Wang Z, Pan Y et al. MiR-26a and miR-26b mediate osteoarthritis progression by targeting FUT4 via NF-ÎºB signaling pathway. Int J Biochem Cell Biol 2018;94:79-88.

# Xie Z, Shen P, Qu Y et al. MiR-20a inhibits the progression of human arthritis fibroblast-like synoviocytes and inflammatory factor expression by targeting ADAM10. Environ Toxicol 2020;35(8):867-878.

# Fan ZD, Cao Q, Huang N et al. MicroRNA-125b regulates Th17/Treg cell differentiation and is associated with juvenile idiopathic arthritis. World J Pediatr 2020;16(1):99-110.

# Xie M, Wang J, Gong W et al. NF-ÎºB-driven miR-34a impairs Treg/Th17 balance via targeting Foxp3. J Autoimmun 2019;102:96-113.

# Dong L, Wang X, Tan J et al. Decreased expression of microRNA-21 correlates with the imbalance of Th17 and Treg cells in patients with rheumatoid arthritis. J Cell Mol Med 2014;18(11):2213-2224.

# Zhang MF, Yang P, Shen MY et al. MicroRNA-26b-5p alleviates murine collagen-induced arthritis by modulating Th17 cell plasticity. Cell Immunol 2021;365:104382.

# Niu Q, Cai B, Huang ZC, Shi YY, Wang LL. Disturbed Th17/Treg balance in patients with rheumatoid arthritis. Rheumatol Int 2012;32(9):2731-2736.

# Li H, Miao D, Zhu Q, Huang J, Lu G, Xu W. MicroRNA-17-5p contributes to osteoarthritis progression by binding p62/SQSTM1. Exp Ther Med 2018;15(2):1789-1794.

# Bosco MC, Delfino S, Ferlito F et al. Hypoxic synovial environment and expression of macrophage inflammatory protein MIP-3a/CCL20 in Juvenile Idiopathic Arthritis. Arthritis Rheum 2008;58:1833-1838.

# Kulshreshtha R, Ferracin M, Wojcik SE et al. A microRNA signature of hypoxia. Mol Cell Biol 2007;27(5):1859-1867.

# Bandara KV, Michael MZ, Gleadle JM. MicroRNA Biogenesis in Hypoxia. Microrna 2017;6(2):80-96.

# Semenza GL. Oxygen sensing, homeostasis, and disease. N Engl J Med 2011;365(6):537-547.

# Bruning U, Cerone L, Neufeld Z et al. MicroRNA-155 promotes resolution of hypoxia-inducible factor 1alpha activity during prolonged hypoxia. Mol Cell Biol 2011;31(19):4087-4096.

# Yang D, Wang J, Xiao M, Zhou T, Shi X. Role of Mir-155 in Controlling HIF-1Î± Level and Promoting Endothelial Cell Maturation. Sci Rep 2016;6:35316.

# He M, Wang QY, Yin QQ et al. HIF-1Î± downregulates miR-17/20a directly targeting p21 and STAT3: a role in myeloid leukemic cell differentiation. Cell Death Differ 2013;20(3):408-418.

# Zhou S, Sun L, Cao C et al. Hypoxia-induced microRNA-26b inhibition contributes to hypoxic pulmonary hypertension via CTGF. J Cell Biochem 2018;119(2):1942-1952.

# Poitz DM, Augstein A, Gradehand C, Ende G, Schmeisser A, Strasser RH. Regulation of the Hif-system by micro-RNA 17 and 20a - role during monocyte-to-macrophage differentiation. Mol Immunol 2013;56(4):442-451.

# Yan HL, Xue G, Mei Q et al. Repression of the miR-17-92 cluster by p53 has an important function in hypoxia-induced apoptosis. EMBO J 2009;28(18):2719-2732.

# Sun KT, Chen MY, Tu MG, Wang IK, Chang SS, Li CY. MicroRNA-20a regulates autophagy related protein-ATG16L1 in hypoxia-induced osteoclast differentiation. Bone 2015;73:145-153.

# Xu Z, Zhao L, Zhu LY, He M, Zheng L, Wu Y. MicroRNA-17, 20a regulates the proangiogenic function of tumor-associated macrophages via targeting hypoxia-inducible factor 2Î±. PLoS One 2013;8(10):e77890.

# Dejean E, Renalier MH, Foisseau M et al. Hypoxia-microRNA-16 downregulation induces VEGF expression in anaplastic lymphoma kinase (ALK)-positive anaplastic large-cell lymphomas. Leukemia 2011;25(12):1882-1890.

# Ortona E, Pierdominici M, Maselli A, Veroni C, Aloisi F, Shoenfeld Y. Sex-based differences in autoimmune diseases. Ann Ist Super Sanita 2016;52(2):205-212.

# Nicolau J, Lequerrè T, Bacquet H, Vittecoq O. Rheumatoid arthritis, insulin resistance, and diabetes. Joint Bone Spine 2017;84(4):411-416.

# Wong SC, Dobie R, Altowati MA, Werther GA, Farquharson C, Ahmed SF. Growth and the Growth Hormone-Insulin Like Growth Factor 1 Axis in Children With Chronic Inflammation: Current Evidence, Gaps in Knowledge, and Future Directions. Endocr Rev 2016;37(1):62-110.

# Sun J, Feng M, Wu F et al. Plasma miR-26a as a Diagnostic Biomarker Regulates Cytokine Expression in Systemic Juvenile Idiopathic Arthritis. J Rheumatol 2016.

# Nziza N, Duroux-Richard I, Apparailly F. MicroRNAs in juvenile idiopathic arthritis: Can we learn more about pathophysiological mechanisms? Autoimmun Rev 2019;18(8):796-804.

# Tang J, Lin J, Yu Z et al. Identification of circulating miR-22-3p and let-7a-5p as novel diagnostic biomarkers for rheumatoid arthritis. Clin Exp Rheumatol 2021.

# Ali SA, Gandhi R, Potla P et al. Sequencing identifies a distinct signature of circulating microRNAs in early radiographic knee osteoarthritis. Osteoarthritis Cartilage 2020;28(11):1471-1481.

# Makki MS, Haqqi TM. miR-139 modulates MCPIP1/IL-6 expression and induces apoptosis in human OA chondrocytes. Exp Mol Med 2015;47:e189.

# Liu J, Jiang Y, Han M et al. MicroRNA-345-5p acts as an anti-inflammatory regulator in experimental allergic rhinitis via the TLR4/NF-κB pathway. Int Immunopharmacol 2020;86:106522.

# Huang Y, Chen K, Yu H et al. Up-regulated microRNA-411 or declined RIPK1 inhibits proliferation and promotes apoptosis of synoviocytes in rheumatoid arthritis mice via decreased NF-κB pathway. Cell Cycle 2020;19(6):666-683.

# Lin Z, Tian XY, Huang XX, He LL, Xu F. microRNA-186 inhibition of PI3K-AKT pathway via SPP1 inhibits chondrocyte apoptosis in mice with osteoarthritis. J Cell Physiol 2019;234(5):6042-6053.

# Yang J, Zhuang Y, Liu J. Upregulation of microRNA-590 in rheumatoid arthritis promotes apoptosis of bone cells through transforming growth factor-β1/phosphoinositide 3-kinase/Akt signaling. Int J Mol Med 2019;43(5):2212-2220.
