# Supplementary material for: Extracellular vesicle‐derived microRNAs as potential biomarkers in oligoarticular juvenile idiopathic arthritis patients: methodological challenges and new perspectives
Source: Clin Transl Med. 2022 Sep 30;12(10):e1067. doi: 10.1002/ctm2.1067 (PMC9523680; doi:10.1002/ctm2.1067)
Supplement: Supplementary file 2 — Supplementary information [file CTM2-12-e1067-s002.docx]

**Supplementary Figures**

**Extracellular vesicle-derived microRNAs as potential biomarkers in Oligoarticular Juvenile Idiopathic Arthritis patients: methodological challenges and new perspectives**

**This file includes:**

| **Supplementary Figures** | **Page** |
| --- | --- |
| **Figure S1.** Flow chart of SF sample processing and EV-miR isolation/analysis in OJIA patients. | 2 |
| **Figure S2.** EV-miR detection in EDTA vs heparin/de-heparinized SF samples from patients of the training cohort. | 3 |
| **Figure S3.** Comparative analysis of EV-miR expression profiles in EDTA respect to heparin/de-heparinized SF samples from patients of the training cohort. | 4 |
| **Figure S4.** Characterization of EVs and the EV small-RNA profile in PL samples from OJIA patients. | 6 |
| **Figure S5**. Validation by qRT-PCR of differentially expressed EV-miRs in OJIA-SF respect to OJIA-PL specimens. | 8 |
| **Figure S6.** Validation by qRT-PCR of differentially expressed EV-miRs in OJIA-SF and OJIA-PL respect to CTR-PL specimens. | 9 |

**Figure S1. Flow chart of SF sample processing and EV-miR isolation/analysis in OJIA patients.** SF samples from 13 new-onset OJIA patients were collected into EDTA and/or sodium-heparin tubes and then split into two aliquots, one treated with HYase (samples 1B, 2B) and the other left untreated (samples 1A,2A). EVs were purified from SF specimens with the exoRNeasy Serum/Plasma kit. Total RNA was extracted from isolated EVs with the exoRNeasy Serum/Plasma kit combined with Qiazol. Aliquots of RNA preparations from samples 2A and 2B were treated with heparinase (samples 2C, 2D). EV-miR expression profile was analyzed by the TaqMan Array Card Technology. Differential expression analysis was carried out on qRT-PCR expression data. Pathway analysis was performed on validated EV-miR targets.

**
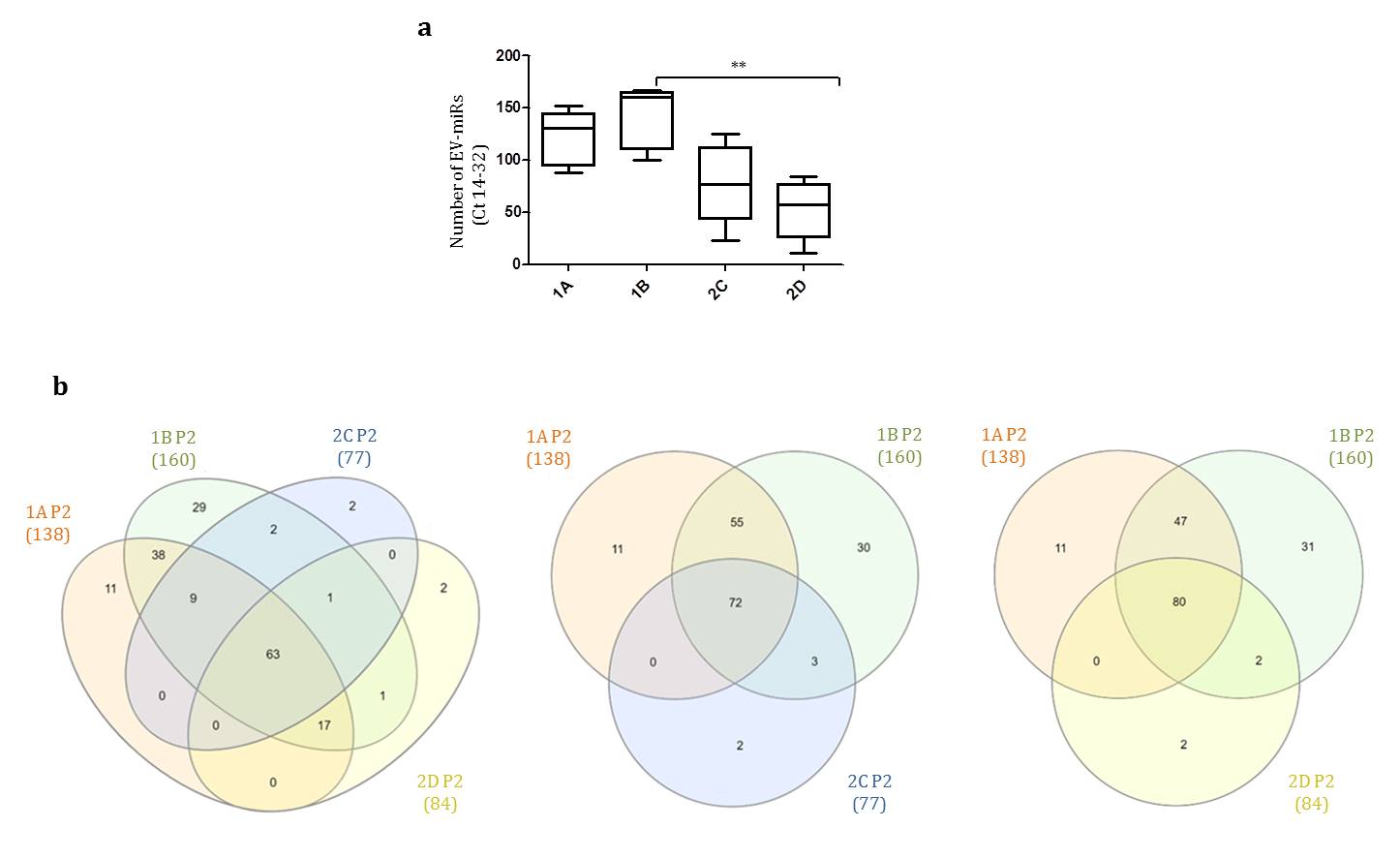
Figure S2. EV-miR detection in EDTA vs heparin/de-heparinized SF samples from patients of the training cohort.** The number of EV-miRs with CT values falling within the range of 14-32 was determined by PIPE-T in SF samples from patients of the training cohort processed as described in the legend of Fig. S1. **(a)** Box plots indicate the average number of detectable EV-miRs in the 5 patients analyzed. Boxes contain the values falling between the 25th and 75th percentiles, horizontal lines represent median values, and whiskers (lines that extend from the boxes) represent the highest and lowest values for each group. Statistical analysis using paired Student’s t-test is performed. p value of 2D relative to 1B: **p < 0.01 **(b)** Venn diagram shows the number of common and exclusive EV-miRs detectable in the samples from a representative patient (P2). Each type of sample processing procedure is depicted by a distinct color. The left diagram represents all treatment conditions; the middle diagram represents EDTA (±HYase) and heparin/de-heparinized samples; the right diagram represents EDTA (±HYase) and heparin/de-heparinized (+HYase) samples.


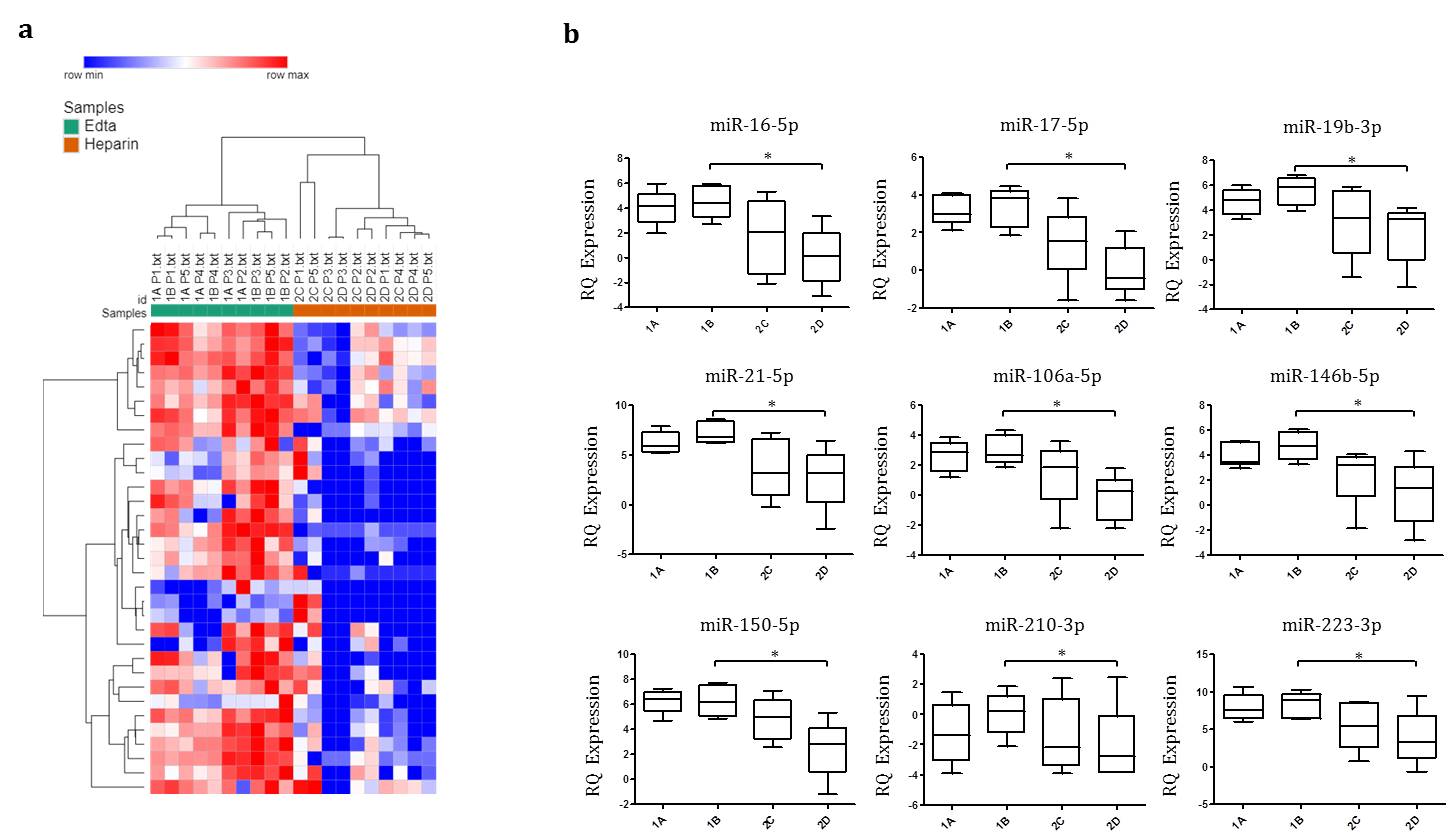


**Figure S3. Comparative analysis of EV-miR expression profiles in EDTA respect to heparin/de-heparinized SF samples from patients of the training cohort. (a)** Heat-map representation and unsupervised hierarchical clustering analysis of differentially expressed EV-miRs among the EDTA (±HYase) and heparin/de-heparinized (±HYase) groups of samples. Data are relative to EV-miR expression in SF from the 5 patients of the training cohort processed as detailed in the legend of Fig. S1. Expression levels were z-scored and log2 transformed and are indicated by a 2-color scale ranging from blue (lowest values) to red (highest values) reported in the horizontal bar at the top of the figure. Each column represents a patient (indicated on the top side) and each row represents an EV-miR. Dendogram reports the results of the unsupervised hierarchical clustering and is displayed in the top of the plot. Two main clusters of patients were identified corresponding to the EDTA and heparin/de-heparinized groups of samples, and are displayed at the top of the plot. **(b)** Box plots show the mean RQ expression of nine representative differentially expressed EV-miRs in SF samples from the 5 patients analyzed. Boxes contain the values falling between the 25th and 75th percentiles, horizontal lines represent mean values, and whiskers (lines that extend from the boxes) represent the highest and lowest values for each group. Data were analyzed with paired Student’s t test and are expressed in log2. p value of sample 2D relative to 1B: *p < 0.05


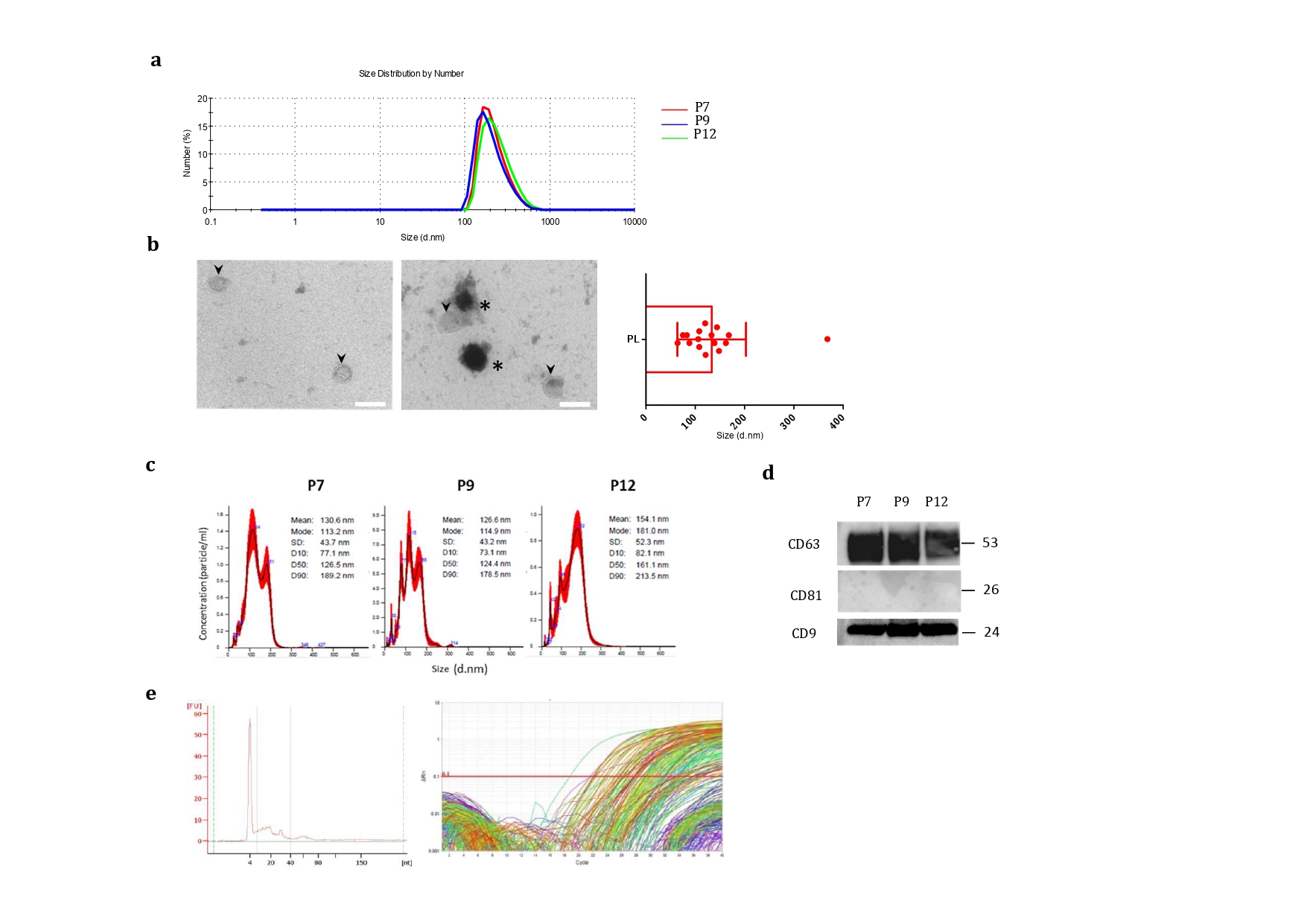


**Figure S4. Characterization of EVs and the EV small-RNA profile in PL samples from OJIA patients.** PL samples from patients of the validation cohort were collected in EDTA tubes, EVs were isolated from 500 μl of sample using the exoRNeasy Serum/Plasma Kit and characterized. Small-RNAs were extracted by exoRNeasy serum/plasma kit combined with Quiazol. **(a)** Curve shows the bell-shaped size distribution profile of EVs isolated from the PL of three patients analyzed in Fig.1b (P7, P9, P12), measured by DLS using the Zetasizer Nano ZS90 particle size analyzer (peaks at 209, 223, 245 nm; PDI 0.14, 016, 0.16). **(b)** Representative TEM images of isolated EVs visualized by negative staining (left and middle panels). EVs showing the typical round ultrastructural morphology are indicated by arrowheads. Dense precipitates are shown by asterisks. Scale bars = 200 nm. Size distribution of isolated EVs analyzed by TEM (right panel) is visualized as detailed in Fig.1c (mean EV diameter 133.4±69.9 nm). **(c)** NTA of EVs isolated from the patients analyzed in panel b and diluted 1:1000 in particle-free PBS. Line graphs indicate the average size of EVs calculated from 5 different measurement for sample. The diameter (nm) of EVs is reported on the X-axis, while the concentration (particles/mL) is reported on the Y-axis. EV size mean and mode are shown. D90 , D50, and D10 refer to the percentage of EVs (90%, 50%, 10%) with size lower than the values indicated. **(d)** Western blot analysis of the whole EV protein extracts in PL samples from the three representative patients analyzed by DLS was performed with the anti-CD9, anti-CD63, and anti-CD81 mAbs, as described in Fig.1e.**(e)** RNA quality and miR content were assessed as described in Fig.1e. The electropherogram (left panel) represents the small-RNA profile of a representative sample. The plot (right panel) represents the qRT-PCR amplification curve of EV-RNA from the PL of a representative patient.

**
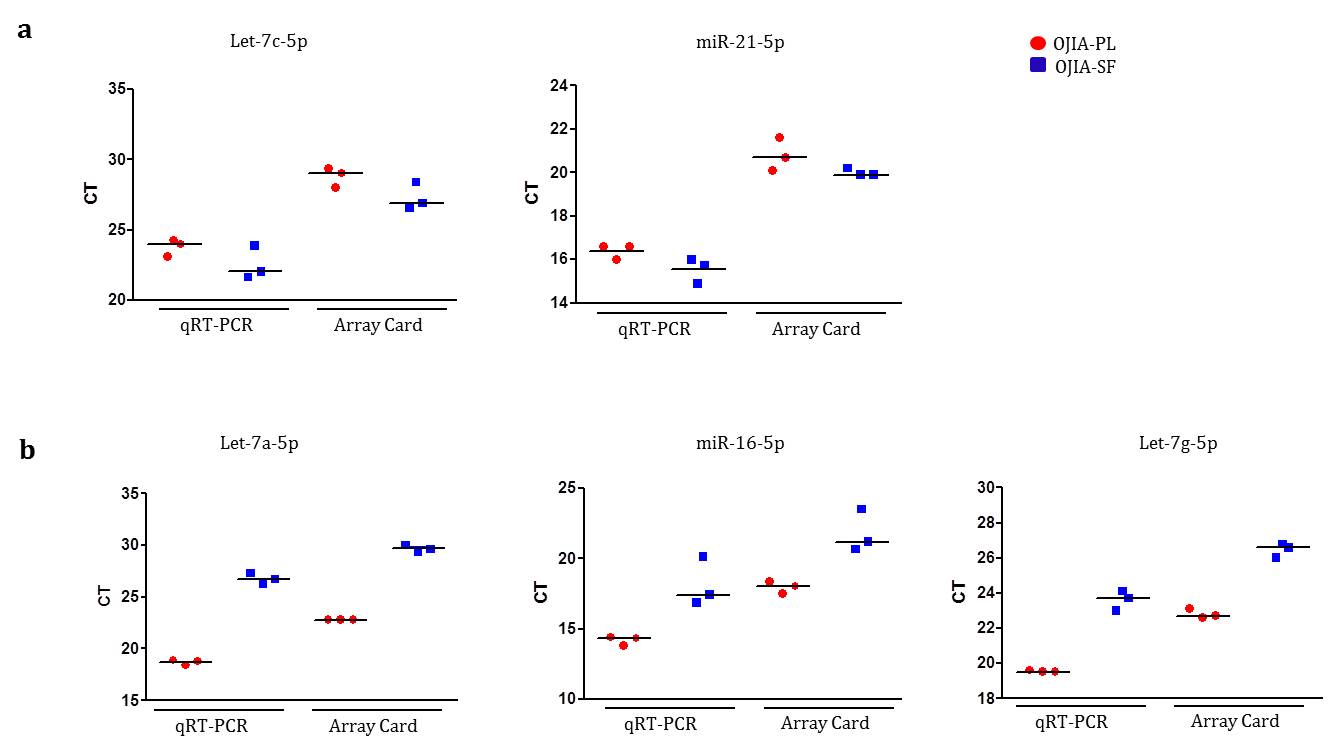
Figure S5. Validation by qRT-PCR of differentially expressed EV-miRs in OJIA-SF respect to OJIA-PL speciments**. Relative expression of let-7c-5p, miR-21-5p, let-7a-5p, miR-16-5p, and let-7g-5p was validated by qRT-PCR in SF and PL samples from three representative OJIA patients using the TaqMan miRNAs Assay. The scatter dot plot represents the mean CT values calculated on the basis of triplicate measurements for each experiment obtained by both Array Cards and qRT-PCR. Each point represents a single patient/control subject. Horizontal lines indicating median values for each group. Each group of samples is depicted by a specific color.


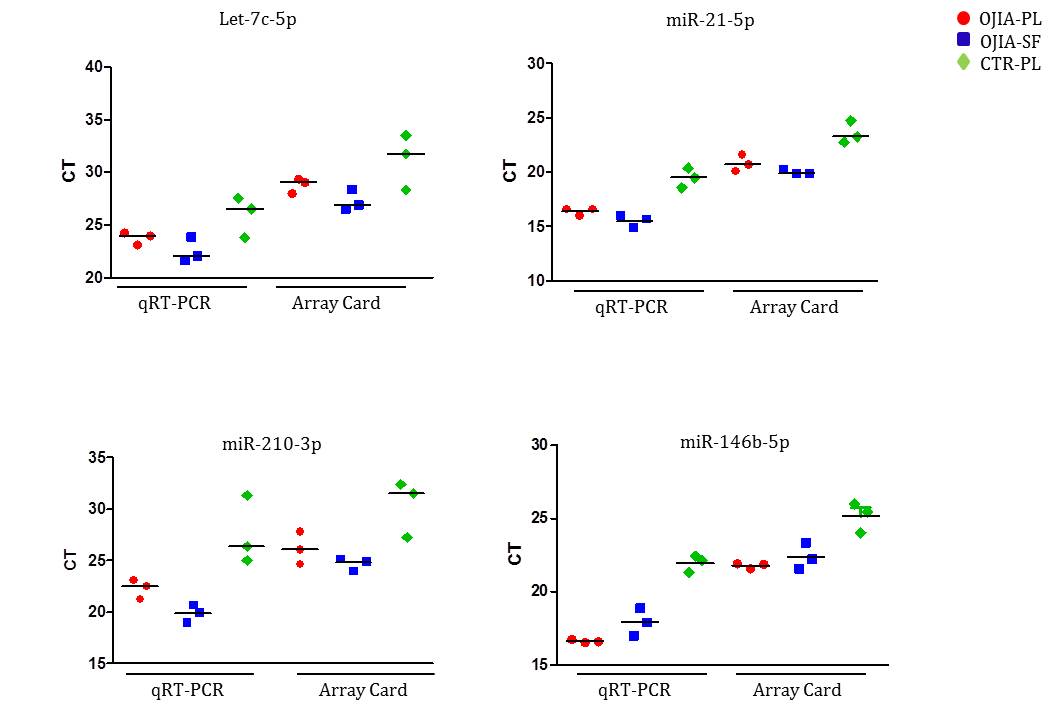


**Figure S6. Validation by qRT-PCR of differentially expressed EV-miRs in OJIA-SF and OJIA-PL respect to CTR-PL speciments.** Relative expression of let-7c-5p, miR-21-5p, miR-210-5p, and miR-146b-5p was validated by qRT-PCR in SF and/or PL samples from three representative OJIA patients and PL from three control children using the TaqMan miRNAs Assay. Data are represented as detailed in the legend of Figure S5.
